# Expectations, experiences and challenges of nursing students using the virtual learning medium during the COVID-19 pandemic: A descriptive phenomenological study

**DOI:** 10.1371/journal.pone.0299967

**Published:** 2024-03-08

**Authors:** Puoza Deo Gracious, Jerry Armah, Edward Appiah Boateng, Victoria Bam, Veronica Dzomeku, Joana Kyei-Dompim, Ampem Darko Oklodu-Abbey, Abigail Kusi Amponsah

**Affiliations:** 1 Department of Nursing, Kwame Nkrumah University of Science and Technology, Kumasi, Ghana; 2 Nursing and Midwifery Council of Ghana, Accra, Ghana; 3 Department of Nursing Science, University of Turku, Turku, Finland; Queensland University of Technology, AUSTRALIA

## Abstract

**Background:**

The COVID-19 pandemic caused several higher educational institutions to switch from traditional face-to-face to virtual learning medium. This abrupt shift came with new expectations, experiences and challenges to nursing/ midwifery students, particularly new users, and even more so when preparation, orientation, and support were lacking or ineffective. The present study therefore aimed at exploring the expectations, experiences and challenges of nursing students using the virtual learning medium during the COVID-19 pandemic.

**Methods:**

This was a descriptive phenomenological design using 12 purposively sampled nursing and midwifery students from a public university in Ghana. With the aid of a semi-structured guide, individual face-to-face interviews were audiotaped, transcribed verbatim at a later time and deductively analyzed into themes using the customer experience execution model.

**Results:**

Participants were aged 22–36 years and involved equal number of males and females (n = 6), with majority being Christians (n = 11). Six themes were generated from the study: (1) “Initial thoughts and emotions” described participants initial reactions to the virtual educational medium during the pandemic; (2) "Expectations with the virtual medium" referred to the participants’ anticipations regarding the convenience offered by the virtual medium; (3) "Experiences with the virtual medium" depicted the participants’ recognition of both positive and negative encounters while using the virtual learning platform; (4) “Evaluation and recommendation” described participants’ reports of meeting expectations and recommendations they made to enhance virtual learning; (5) "Challenges and limitations of the virtual medium" typically represented the obstacles encountered by nursing/ midwifery students when they embraced the virtual medium; (6) “Prospects of the virtual medium” referred to participants’ views on the future of the virtual medium.

**Conclusion:**

The study has brought to light that the virtual education environment comes with its own expectations, experiences and challenges to students. Provision of adequate support such as orientation and simulation laboratories by higher education institutions to satisfy students’ needs is necessary to enhance nursing education.

## 1. Introduction

COVID-19 has disturbed all facets of human life as a public health emergency and a worldwide pandemic, with potentially disastrous economic, political, and socioeconomic ramifications [1]. The safety measures to mitigate its spread have provoked widespread societal disruption with recursive impacts within higher educational institutions [2]. Consequently, higher educational institutions were compelled to switch from the face-to-face medium to the virtual learning modes. New forms of online instructions were introduced where the delivery method changed to either synchronous or asynchronous modalities [3].

According to the Cambridge dictionary, virtual learning refers to a system of teaching and learning that uses the internet and special software. Virtual learning is still developing in Africa, and it continues to be a fascinating issue for educators and researchers [4]. In Africa, the transition from the traditional face-to-face to online teaching and learning during the pandemic was problematic as only 24% of the population have access to internet coupled with poor connectivity, high cost of internet bundle and frequent power outages [5]. To ensure smooth and a highly effective virtual medium education, these challenges had to be addressed. However, there was little success in the several initiatives rolled out by the government of Ghana prior to the pandemic to ensure quality and accessible ICT education for all [6]. As a result, several students still struggle with information technologies even at higher educational levels in Ghana. The sudden transition to virtual teaching and learning came as a challenge to several tertiary level students even though most universities already combined both the online learning and the face to face approach [3].

In the study by Bdair [7], nursing students encountered challenges such as lack of training, inadequate network coverage as well as poor connectivity issues especially for those in rural areas. There were issues of lack of motivation and lack of well-established student assessment tools. A study conducted in Canada revealed that while many students appreciated the flexibility and safety provided by online learning, they also reported challenges such as lack of support and a necessity for instructors to receive training in the effective delivery of online classes [8]. Likewise, a study in Ghana showed that while students were initially optimistic about the shift to online learning, expecting greater flexibility and access to resources, they faced realities such as inadequate internet infrastructure, high data costs, and a lack of interactive teaching methods [6]. Marshall and Wolanskyj-Spinner [9] reported that classroom learning is superior to online learning in terms of interaction and clarification. Considering the practical nature of nursing education, there was the challenge of teaching practical competencies online. Inspite of the challenges, online teaching and learning is touted as an important learning strategy as it provides students with flexible learning environment and students have control over time and place for studying [10]. It also has the advantages of remote delivery of learning materials, comfort, accessibility, and easy management [11].

Following the pandemic, some universities in Ghana adopted the virtual learning medium. The traditional way of learning which included lectures in a classroom, demonstration room, laboratories, and clinical settings between instructors and students was the status quo before the pandemic. The sudden shift to the virtual mode presented a lot of uncertainties on the part of students and instructors. While previous studies have touched on general challenges faced by students in general (like connectivity issues, lack of motivation, and need for instructor training), there seems to be a lack of in-depth, qualitative exploration of how nursing students in Ghana, specifically, are navigating the practical aspects of their education in a virtual environment. Nursing education typically involves a significant practical component, and the shift to online learning raises unique challenges in delivering and acquiring practical skills. It is against this backdrop that the current study sought to explore nursing students’ expectations, experiences, and challenges with using the virtual medium for educational purposes. The findings will be significant for educational policymakers, instructors, and curriculum developers in understanding and addressing the unique needs of nursing students in virtual environments.

## 2. Methods

### 2.1 Theoretical framework

**Fig 1:**
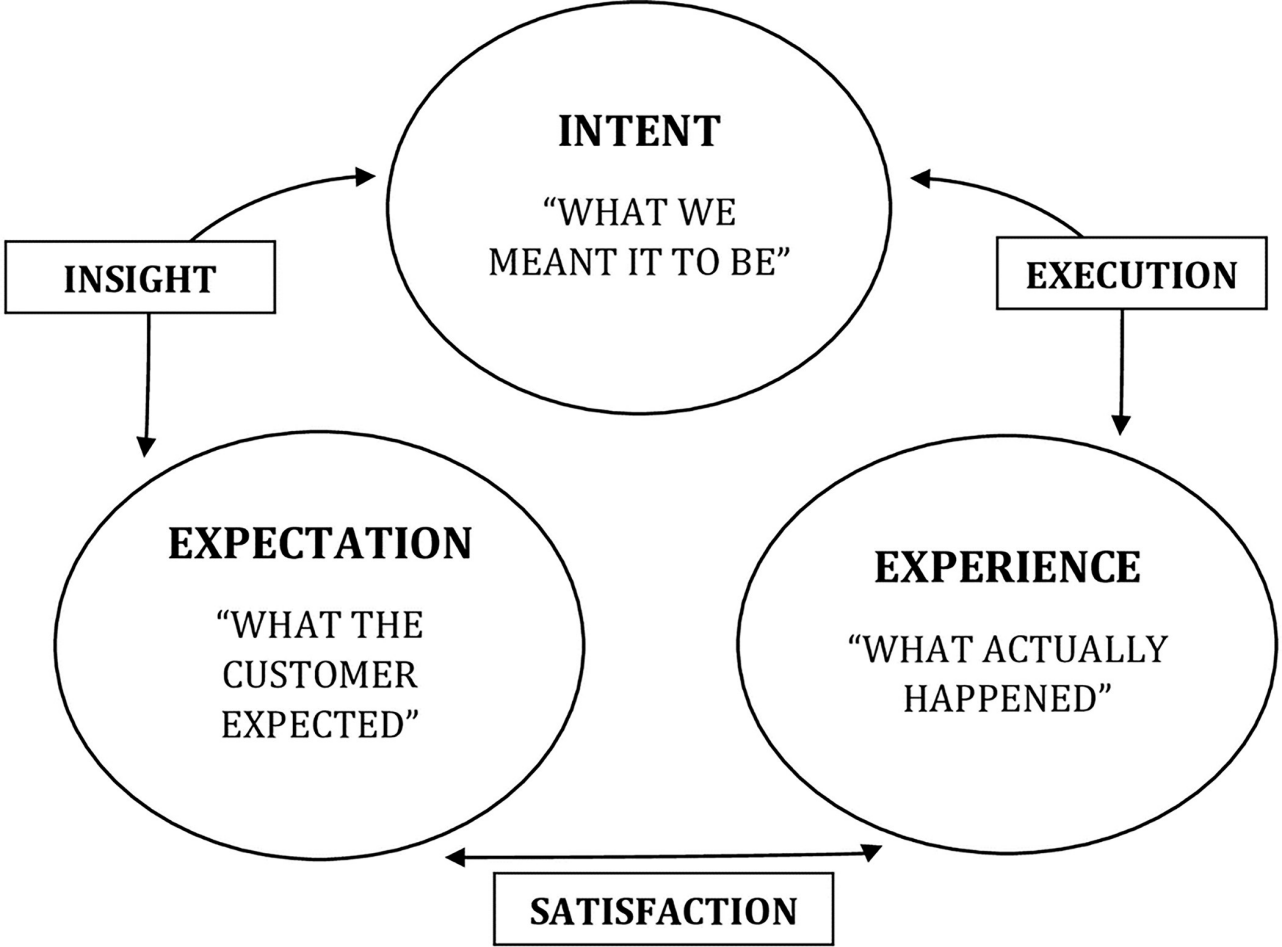
The customer experience execution model.

Customer experience execution (CEE) model framework by Tiersky [12] was used to guide the study. This model is a useful framework for comprehending the various elements that play a role in shaping how users of a digital product or service perceive their digital experiences. As illustrated in Fig 1, the model consists of three constructs namely: intent (what is meant to be), expectation (what is expected) and experience (what happen in reality). Each of these constructs have a relationship with the other and thus can exert positive or negative influences. Whereas positive influences result in “insight”, “execution” and “satisfaction”, negative influences result in gaps or challenges in these areas. Earlier studies have been conducted to examine the specifics of users’ or customers’ digital journeys and provide solutions that boost customer satisfaction [13–15].

### 2.2 Study design

A descriptive phenomenological approach was used since the purpose was to gain a deeper understanding of the participants’ lived experiences, expectations, and challenges regarding their use of the virtual educational medium during the COVID-19 pandemic [16]. The study began on 20^th^ November 2021 and ended on 17^th^ September 2022.

### 2.3 Setting

The research was carried out at the nursing department of the second largest public university in Ghana and provides high-quality education to both local and international students [17]. The nursing department provides training in a wide range of nursing and midwifery programs at both the undergraduate and postgraduate levels. It also offers these programs on a regular and sandwich basis. The regular students attend on a full-time basis comprising of two-semesters within a year, whilst the sandwich students attend on a part-time basis comprising of one-semester within the year. The university was selected due to its wide range of programs serving many students within the country and beyond.

### 2.4 Participants, sampling, and sample size

Inclusion criteria for participation were undergraduate and postgraduate nursing and midwifery students using the virtual medium during the COVID-19 pandemic. Participants were purposively sampled using maximum variation techniques focusing on the different academic levels, genders, and study programs. Sample size was determined by data saturation, where no new insights were generated from the interviews [18].

### 2.5 Data collection and analysis

Prior to data collection, administrative and ethical approval were respectively obtained from the Dean of Students and the Committee on Human Research, Publications and Ethics, School of Medicine and Dentistry, Kwame Nkrumah University of Science and Technology, Kumasi-Ghana. Subsequently, the principal investigator (PDG) approached the eligible students in their classrooms and informed them about the purpose and scope of the project. Interested students provided their details and were contacted at a later time to agree on the date, time, and venue for the face-to-face interviews. A written consent was obtained from participants and recruitment was between 18^th^ January 2022 and 10^th^ February 2022.

Data collection and analysis occurred concurrently for a period of two months. The study tool was pre-tested with three (3) nursing students at a similar university to obtain feedback on its clarity, comprehensibility, and relevance. Based on the feedback obtained, the instrument proved to be appropriate in exploring the phenomenon under study.

**Fig 2:**
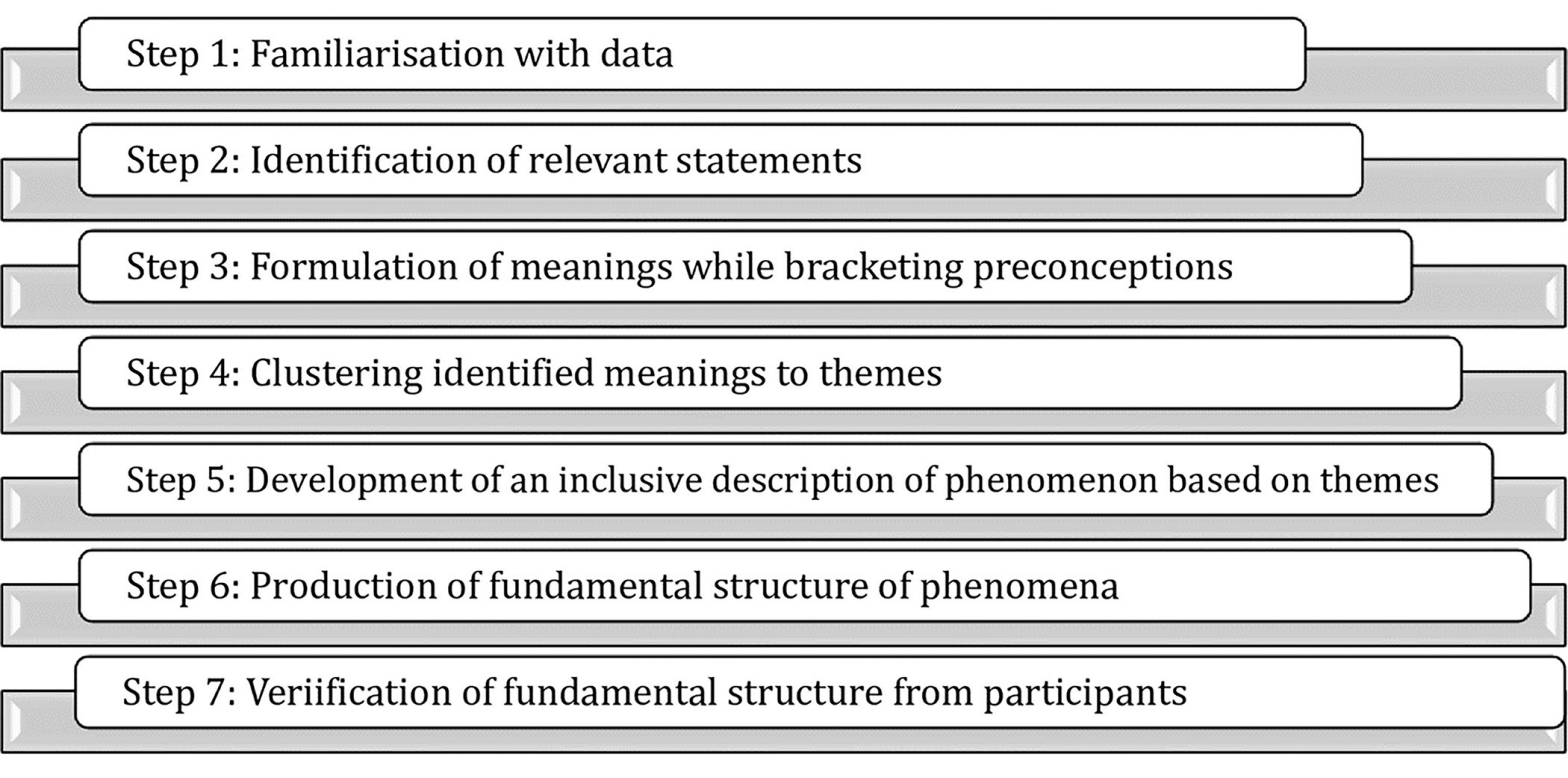
Stages of Colaizzi’s descriptive phenomenological analysis.

The COVID-19 protocols for reducing viral transmission such as wearing of nose mask, social distancing and the use of hand sanitizers were followed throughout the interviews. The interviews were facilitated by the principal investigator who is a mixed methods researcher with five years in qualitative research, and an assistant who took field notes and also recorded the interviews with participants’ permission using an audio recorder. A semi-structured interview guided by the CEE model and the study objectives was used in collecting data. Each interview lasted for 45–60 minutes, were transcribed verbatim at a later time and deductively analyzed with NVivo version 11 into themes using the CEE model (Refer to **Fig 1**) and Colaizzi’s descriptive phenomenological analysis [19] as indicated below in Fig 2. Initially, the researchers familiarized themselves with the data by reading the transcripts to establish a foundational understanding. They then extracted key statements that captured the essence of the students’ experiences. These statements were carefully interpreted, with the researchers setting aside their preconceived notions to prevent bias. The interpreted meanings were clustered into themes that reflected the core aspects of the experiences described by the participants. With these themes, a detailed narrative was crafted, presenting a comprehensive view of the phenomenon. This narrative was refined into a fundamental structure that represented the true nature of the students’ virtual learning experience. Finally, this structure was presented back to the participants for validation, ensuring that the findings were a true reflection of their lived experiences.

### 2.6 Ethical considerations

Ethical clearance was obtained from the Committee on Human Research and Publications Ethics (CHRPE/AP/542/21) in Kwame Nkrumah University of Science and Technology (KNUST), Ghana before commencing data collection. The participants’ written informed consents were obtained, and every aspect of the study was described to them in detail. Participants’ right to be self-determined (autonomy) and voluntary participation were respected throughout the study. The data collected was anonymized and treated confidentially.

### 2.7 Trustworthiness

Lincoln and Guba [20] evaluative criteria were employed to ensure trustworthiness in the study: credibility, transferability, dependability, and confirmability. In this study, credibility was established by providing participants with comprehensive details about the study’s objectives and procedures, building a good rapport before the interviews, reflecting on the researchers’ influence on the research processes, memoing and bracketing any preoccupations. To ensure transferability, a thorough description of the setting, participants, data collection methods, analysis and findings was provided. With regards to dependability, consistency was maintained in the interview approach by using the same researcher to facilitate all the interview sessions, recording all interviews and the use of verbatim transcription. Confirmability was achieved by data saturation (when no new information or themes were generated from the interviews, indicating the collection of sufficient data), member checking (providing participants with the opportunity to review and verify the accuracy of interview transcripts and summaries) and triangulation, which involved using multiple data sources (interviews and field notes) and researchers to enhance the interpretations derived from the data (peer debriefing).

## 3. Results

### 3.1 Sociodemographic characteristics of participants

Study participants were six (6) males and six females (6) who were aged 22–36 years. Many of the participants (8/12) were nursing students pursuing undergraduate General and Emergency Nursing, as well as postgraduate Nursing programs. Participants’ year of study ranged level 300 (third year) to level 600 (sixth year), and almost all of them (11/12) were Christians (Refer to Table 1).

**Table 1:** Participants’ demographic data.

Participant ID	Gender	Age (years)	Program	Level	Religion
NS001	Female	24	BSc. Nursing	300	Christianity
NS002	Male	32	MPhil. Nursing	600	Christianity
NS003	Female	27	BSc. Emg. Nursing	400	Christianity
NS004	Female	25	BSc. Midwifery	300	Christianity
NS005	Female	26	BSc. Midwifery	300	Christianity
NS006	Male	22	BSc. Nursing	300	Christianity
NS007	Male	23	BSc. Emg. Nursing	400	Christianity
NS008	Female	23	BSc. Midwifery	300	Christianity
NS009	Female	24	BSc. Midwifery	400	Christianity
NS010	Male	29	BSc. Nursing	400	Christianity
NS011	Male	27	BSc. Nursing	300	Muslim
NS012	Male	36	MPhil. Nursing	500	Christianity

**Note:** ID–Identification, BSc.–Bachelor of Science, MPhil.–Master of Philosophy, Emg.–Emergency.

### 3.2 Themes

**Fig 3:**
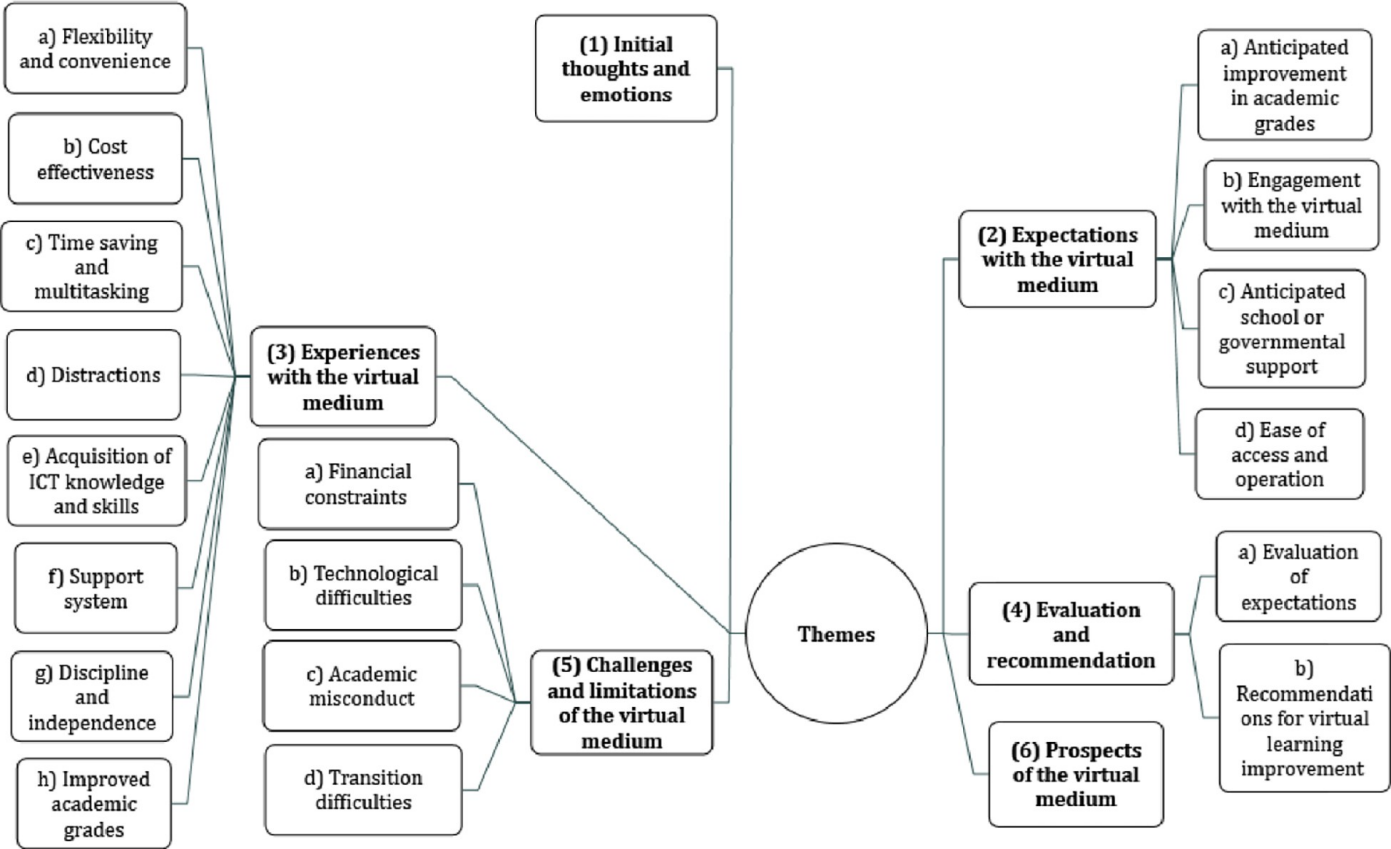
Diagrammatic representation of study themes and subthemes. (Main themes are represented by numbers (1–5) and corresponding subthemes are represented by alphabets under each main theme.

Based on the CEE model [12] that guided the study and Colaizzi’s descriptive phenomenological analysis [19], six (6) themes and eighteen (18) subthemes emerged from the study as presented below in Fig 3.

### 3.3 Initial thoughts and emotions

This theme explored participants’ reaction to the virtual medium when it was first rolled out. According to participants, there were a mixture of feelings from excitement to nervousness at the beginning of the virtual medium usage. This was expressed in their quotes as:

"*Although I was happy*, *I also felt that this pandemic will distract our academic calendar*.*"****NS005***

"*Initially*, *I had mixed feelings about it*. *I was excited I could continue my education while at home but at the same time worried about how to use the virtual medium like the zoom app*.*"* ***NS009***

Another participant also stated:

"*Initially at the beginning I was nervous because it was a new thing introduced to us*.*"****NS010***

### 3.4 Expectations with the virtual medium

This theme described participants’ anticipation about possible support from the institution and government with the use of the virtual medium. They exhibited intentions to continue their academic work and improve their academic performance and grades when they described their hopes for using the virtual medium of education. Four (4) subthemes emerged from this theme: anticipated improvement in academic grades, engagement with the virtual medium, anticipated school or governmental support, ease of access and operation.

#### 3.4.1 Anticipated improvement in academic grades

The majority of participants said that they anticipated the virtual medium to aid in the improvement of their grades. Participants opined that there was an ease with taking quizzes and assignments, which was expected to improve their grades. They stated:

“*I expected my grades to improve using the virtual medium*.*"* ***NS004***

"*I expected my grades to improve because taking a quiz or an examination or doing an assignment was very easy as compared to the traditional face-to-face*.*"* ***NS006***

"*Expected my grades to be better because the virtual medium offers me the opportunity to revisit what was taught previously*.*"* ***NS012***

#### 3.4.2 Engagement with the virtual medium

Participants reported that the virtual medium had the potential to improve the active engagement of both students and lecturers. On the part of students, they indicated how they could overcome shyness and be active during class sessions. They also reported how they expected lecturers to be as active as they were during the traditional face-to-face. This was evident in their quotes:

"*I am a shy person and so I do not like contributing to the physical class due to the physical presence of colleagues*. *But with zoom*, *I could actively partake in the virtual class by answering and asking questions*.*"* ***NS001***

"*I also expected a follow-up by lecturers on how the assignment was done using the virtual medium*.*"* ***NS004***

"*I expected to see my lecturers deliver as they did in the traditional face-to-face*. *I expected to hear them very clearly using the virtual medium just as we have in the traditional face-to-face*.*"* ***NS006***

#### 3.4.3 Anticipated school or governmental support

This subtheme described how participants expected the government and school to support them as necessary for an active utilization of the virtual medium. This support was mainly in the form of access to the internet such as data packages. Participants stated:

"*I was expecting the government or the school management to support us adequately during these trying times of covid-19 pandemic so far the virtual learning is concerned*.*"* ***NS001***

"*I was expecting that the university will bear the cost of data for us*.*"****NS004***

#### 3.4.4 Ease of access and operation

The ability to access the virtual medium with ease, and also without internet was evident in the reports of participants. They reported how they anticipated to use the virtual medium with less difficulties. This was evident in their quotes as:

"*I also thought some apps could be operated offline or used without the internet*. *But I realize that so much data was needed to operate them*.*"* ***NS008***

"*I also expected to be able to use the virtual medium with ease*.*"* ***NS011***

### 3.5 Experiences with the virtual medium

This theme described participants’ acknowledgment of both favorable and unfavorable experiences when using the virtual learning medium. Participants had different opinions about their interactions with the virtual medium. Eight (8) subthemes emerged from this theme: flexibility and convenience, cost effectiveness, time saving and multitasking, distractions, acquisition of ICT (information and communication technology) knowledge and skills, support system, discipline, and independence, and improved academic grades.

#### 3.5.1 Flexibility and convenience

Participants shared their experiences on the flexible and convenient nature of the virtual educational medium. This was mainly because they could be available for lectures in the comfort of their homes, and able to combine work with classes effectively unlike the traditional face-to-face. They made comments such as:

“*Opportunities in a sense that you are able to combine your work schedule and be able to school at the same time*, *unlike in the past where you will move from my location to another to be able to have your education*. *So that’s one positive impact of coverage on my learning*.*"* ***NS002***

"*For example*, *I could be studying while in a car or the kitchen*.*"* ***NS003***

"*It’s been beneficial because of how cost-effective*, *convenient*, *and flexible it is*.*"* ***NS010***

#### 3.5.2 Cost-effectiveness

The participants claimed that the expense of attending school on campus was avoided. They talked about how this experience improved their savings and helped them cut costs. They also indicated how they did not have to spend much money for some basic necessities such as food since they were at home. They made comments such as;

“*For instance*, *I work at Tamale in the northern part of Ghana*, *so if not with the virtual medium*, *it means I needed to travel from Tamale to Kumasi to have lectures*. *So*, *with this virtual*, *it has saved me time and resources from traveling up and down*.*"* ***NS002***

"*I didn’t need to go to campus for lectures and so I did not need to buy food*, *pay for accommodation*, *and transport fairs*.*"* ***NS006***

#### 3.5.3 Time-saving and multitasking

Several participants acknowledged that they were able to successfully manage their time while studying from home using the virtual environment. They revealed that pre-recorded lectures gave them the chance to make good use of their time and make time for other extracurricular activities. This helped them develop some time management skills.

"*I had to audio record the lecture whilst at the funeral grounds*, *and this enabled me to succeed with the two events concerning managing my time*.*"* ***NS003***

"*I was also able to manage my time very efficiently because the virtual medium is very flexible*, *and so I could multitask during the lockdown whilst using the virtual medium*.*"* ***NS005***

“*I am also able to manage my time very well as I used the virtual medium*.*"* ***NS006***

#### 3.5.4 Distractions

Some participants admitted they lost track of how many lectures they had to miss due to distractions. According to participants, these distractions were of several kinds such as domestic and using phones for other purposes whiles class was in session. The participants’ descriptions of the distraction in their homes were very diverse in nature. The quotes below attest to this:

"*For example*, *during zoom lectures*, *I may be receiving calls*, *WhatsApp messages*, *or even playing music among others*.*"* ***NS001***

"*Sometimes there are things I am supposed to understand*, *but I don’t do it as home activities do not allow me to concentrate when using the virtual medium*.*"* ***NS003***

"*Most of us did not concentrate in the virtual classes as students were engaged in other activities such as WhatsApp chatting*, *playing music*, *etc*.*"* ***NS006***

#### 3.5.5 Acquisition of ICT knowledge and skills

Participants described how using virtual platforms on electronic devices helped them improve upon their ICT knowledge and abilities, especially in this digital age. The following stories were shared by participants to illustrate the benefits that the virtual medium provided to them:

"*I was able to acquire some skills in ICT*. *I am now able to use these ICT tools like laptops and the virtual medium software with little difficulty*.*"* ***NS001***

“*The persistent use of electronic devices and virtual platforms helped me to acquire some ICT skills*.*"* ***NS010***

**"***I was able to get some skills with the virtual medium using my computer and other ICT gadgets*.*"* ***NS011***

#### 3.5.6 Support system

Participants described the various support systems that were available as they used the virtual medium. They acknowledged the university’s support in the form of the provision of data bundle subscriptions, free Wi-Fi, ICT laboratory, and technical support from ICT personnel. Some of their quotes are:

"*ICT experts were readily available to assist us with any challenges we may encounter with our virtual learning activities*.*"* ***NS003***

"*The university provided us with a data package to support us in our virtual learning*.*”* ***NS006***

"*Well*, *for me*, *when I went back to campus I was able to make good use of the ICT laboratory as well as the Wi-Fi which helped me a lot*.*"* ***NS011***

Also, according to some participants, they received support primarily from their immediate family members, particularly the parents, who made the necessary provisions like a laptop and other necessary technological gadgets for learning. They also revealed how friends occasionally supported technically with the virtual medium of education. Some of the accounts are:

"*My parents bought me a router following the challenges I had with my internet connectivity so with a router I could enjoy my Virtual class without any difficulty or distraction*.*"* ***NS004***

"*A laptop I borrowed from a friend helped me to participate in the virtual medium*.*"* ***NS007***

"*My parents bought me a phone and a new laptop for this virtual medium*.*"* ***NS008***

#### 3.5.7 Discipline and independence

Participants revealed that the virtual medium aided in the development of an attitude of discipline and independence. According to participants, the medium helped them to be self-motivated and become independent learners. This was evident in their quotes as:

"*But with the covid-19 emergence and the use of the virtual medium*, *I had to make independent learning and discipline part of me*. *And this helped me*.*"****NS001***

"*With the virtual medium*, *I’ve been able to acquire some attitude toward learning independently of my colleagues and lecturers because it is a self-paced learning medium*.*"* ***NS011***

"*It also made me develop an attitude of independent learning*.*"* ***NS012***

#### 3.5.8 Improved academic grades

Majority of the participants reported that they observed an improvement in their grades after adopting the virtual medium. They indicated that even though the abrupt shift to the virtual medium was unexpected, it was still better because they could improve their grades. Some reported that the suboptimal ability of the virtual medium in assessing students adequately might have contributed to the rise in the academic grades of students. Some of their quotes are:

"*Despite the sudden shift from the traditional face-to-face to the virtual medium*, *my grades were very good*. *My grades were better compared with the traditional face-to-face medium*.*"* ***NS001***

"*My grades improved so much but I think it was all because of how loose the virtual medium was concerning assessing the students*.*"* ***NS008***

"*My ability to audio record and listen to lectures later improve my grades tremendously*.*"* ***NS009***

### 3.6 Evaluation and recommendation

This theme emerged from participants descriptions on the extent to which their expectations were met, as well as some recommendations for a better virtual medium learning experience. Two subthemes emerged from this theme: Evaluation of expectations and Recommendations for virtual learning improvement.

#### 3.6.1 Evaluation of expectations

Participants reported that their expectations were partially met. None of the participants indicated that that expectations were fully met. They consistently reported a partial success rate of the virtual medium. This was evident in their quotes as:

"*Yes*, *I can say to some extent some expectations have been met because I was able to acquire the necessary skills that came with the virtual medium*. *However*, *the quality of delivery and assessing students were not met at all*.*"* ***NS002***

"*Well*, *I can say my expectations as an outline to you were partially met*.*"* ***NS008***

"*My expectations were partially met as I could not see my lecturers fully*.*"* ***NS011***

#### 3.6.2 Recommendations for virtual learning improvement

This subtheme emerged from recommendations made by participants to improve virtual medium learning in the future. These recommendations were diverse from institutional training and support to governmental support. They made some recommendations such as:

"*To begin with*, *the university management needs to train both students and lecturers on the use of the virtual medium*.*"* ***NS008***

"*Government should invest in the virtual medium so that institutions can fully adopt it into their curricula*.*"* ***NS009***

"*The government of Ghana must put in place structures to have well-established ICT centers in all instances of higher learning across the country*.*"* ***NS010***

### 3.7 Challenges and limitations of the virtual medium

This theme typically describes the difficulties faced by nursing/ midwifery students when they adopted the virtual medium and its associated limitations. Four subthemes were generated from this theme namely: financial constraints, technological difficulties, academic misconduct, and transition difficulties.

#### 3.7.1 Financial constraints

Participants recounted some financial constraints which accompanied the transitioning to the virtual educational medium during the pandemic. For a number of them, they had not anticipated how costly data bundles and other virtual learning equipment were. Participants’ comments are represented by the following quotations:

"*Negatively*, *I can say that the data bundle was too costly for me*.*"* ***NS001***

"*It was very costly for me to get data*. *I also faced tough difficulties acquiring the appropriate electronic devices such as laptops*, *smartphones*, *and modems because of my low socioeconomic background*.*"* ***NS002***

"*Economically*, *I could not purchase data for my virtual learning activities during the covid-19 pandemic*.*"* ***NS003***

#### 3.7.2 Technological difficulties

Some participants struggled with poor internet connectivity based on their location. Coupled with these difficulties were the erratic power outages as evidenced in participants’ quotes:

"*Erratic power supply affected my virtual learning so much that any time there was a power outage my virtual learning gets truncated*.*"* ***NS003***

"*Most of the time*, *my poor network did not allow me to have a good virtual medium education experience*.*"* ***NS004***

"*On this fateful day*, *I left home in search of a good network for my virtual class*. *I finally found a place with a good network and in the process of the virtual learning*, *the rain came the whole and so my virtual learning did not happen that day*.*"* ***NS005***

#### 3.7.3 Academic misconduct

Participants revealed that the virtual medium had some limitations such as making students lazy and absenting themselves from class. They also described that since there was no stringent supervision, students engaged in academic dishonesty such as copying quizzes and assignments. This was evident in their quotes as:

"*Most students copied assignments and quizzes and past them on as their own whilst using the virtual medium*.*"* ***NS003***

"*Absenteeism is an order of the day with the virtual medium than the traditional face-to-face*.*"* ***NS005***

“*On the side of the students*, *laziness and lack of discipline are a great distraction for some of us during virtual learning*.*"* ***NS010***

#### 3.7.4 Transition difficulties

Participants reported that the transition to the virtual medium was accompanied with a disadvantage especially for those who could not learn on their own. They described how studying alone at home was challenging and that they felt that they were not ready for the transition. They made comments such as:

"*The virtual medium was adopted as an emergency intervention following the covid-19 pandemic*. *So*, *we the students were not ready for it*.*"* ***NS007***

"*Studying alone in the house was very difficult for me*.*"* ***NS008***

"*Individual learning at the same time when was adopted tax me*, *especially at the beginning*.*"* ***NS009***

### 3.8 Prospects of the virtual medium

This theme was generated from the participants’ views on the future of the virtual medium. Participants described the potential of the virtual medium to continue beyond the pandemic, indicating its advantages and disadvantages. They reported that the virtual medium was here to stay and had potential for the future provided it attracts the necessary support from the necessary stakeholders. Some of their quotes are:

"*I think the virtual medium of education is here to stay whether we like it or not*. *Its advantages of convenience*, *flexibility*, *and cost-effectiveness make it appropriate for both students and instructors*. *I think this is only possible if the government of Ghana is committed to its digital agenda*.*"* ***NS002***

"*I see the virtual medium as having a better future in Ghana*. *However*, *the government and the University management must have to intervene with the issues of the internet connectivity*, *power supply data bundle*, *and access to ICT gadgets for students*.*"* ***NS004***

"*The future is exciting with the virtual medium in Ghana*. *The flexibility*, *convenience*, *and ease of use make the virtual medium a preferred choice for students*.*"* ***NS006***

## 4. Discussion

The current study aimed at exploring the expectations, experiences and challenges of nursing/ midwifery students using the virtual learning medium during the COVID-19 pandemic. The students in this study, who are accustomed to traditional learning expressed a mixture of feelings regarding this sudden change. Participants entered the virtual environment with a variety of expectations including improvement in academic results, student and lecturer engagement, governmental and institutional support, and ease of access and operation. This result is consistent with earlier research that revealed that the primary goal of nursing students is to have a flexible and easy learning environment that will improve their learning and academic success [21,22]. Again, because the virtual medium is versatile and provides some degree of autonomy, participants shifted to it in anticipation of improved academic performance. This is consistent with past research where nursing students anticipated higher academic grades to meet their academic needs [23,24]. However, the results of a study revealed that nursing students often feel disappointed when their expectations for better academic performance are not met. This dissatisfaction leads them to seek alternative teaching methods and strategies [25]. It is therefore believed that as nursing students’ grades improve with the usage of the virtual medium, they are more likely to accept its incorporation to the educational system.

Participants found the cost-effectiveness of the virtual medium to be advantageous. This result is consistent with past research in which nursing students noted how cost-effective using the virtual medium was [26]. However, the majority of participants did have trouble purchasing internet data. This result contradicts the findings of Zarei and Mohammadi [27], who reported that students in high-income nations like the United Kingdom, United States of America, and Germany, were not concerned about data acquisition-related challenges mainly because of wider internet accessibility and relatively cheaper data bundle packages. This inconsistency may be due to the fact that majority of participants of this current study may have come from relatively low socio-economic families in Ghana, hence the reduced financial ability to purchase data. Moreover, internet data bundles are generally not cheap, even for the working class.

Participants also expressed excitement about using the virtual medium since it allowed them to better manage their time than using the traditional face-to-face method while in school. This is in line with the findings of Khalil, et al. [28], who found that nursing students were generally satisfied with the way the virtual learning environment helped them manage and save time. This means the use of the virtual medium allowed participants to have more flexibility in their learning schedule, which is particularly beneficial for individuals who are balancing their education with other commitments. These findings highlight the potential advantages of virtual learning in terms of time management and its positive impact on student satisfaction. The findings from the study showed that nursing students were able to multi-task whilst using the virtual learning medium. This finding is supported by other studies where the nursing students experienced multi-tasking whilst using the virtual learning medium [29]. Even though multitasking can be beneficial, it frequently has negative effects as well, including increased error risk, decreased productivity, and increased stress levels [30].

Furthermore, the study suggests that the use of virtual platforms on electronic devices has had a positive impact on the participants’ ICT knowledge and abilities. This is consistent with findings by Adarkwah [6] and Samaniego Erazo, et al. [31] that emphasize the importance of integrating technology into education to enhance digital literacy and skills in today’s technologically driven world. This indicates that the virtual learning environments and electronic devices can empower students to develop technical competencies and adapt to the digital landscape.

The findings of the current study, however, suggest that there might be some gaps (based on CEE model) between what some participants expected from the online learning experience and what was actually delivered. This could be attributed to various factors such as technical challenges, inadequate support systems, or issues related to communication and engagement in the virtual environment. To enhance the virtual learning experience and meet participants’ expectations more effectively, it is essential to carefully analyze the areas where the virtual medium falls short and make improvements accordingly. This may involve providing additional training and support for both students and instructors, addressing technical issues promptly, and fostering a sense of community and engagement in the virtual classroom [32,33].

Despite the reported positive outcomes, the virtual learning environment also had significant drawbacks for nursing students, such as distractions, financial constraints, academic misconduct, and transition difficulties. Participants reported having an unfavorable home environment while using the virtual learning tool. Similar findings were made by Hensley, et al. [34], who discovered that nursing students encounter distractions in their homes while participating in virtual learning. Participants embraced the virtual learning environment but unwanted interruptions, and other domestic occurrences hindered their progress. Again, an unconducive home environment can lead to students’ dropout from the virtual learning medium and subsequently affect their academic work [35]. Ensuring a conducive home environment through family support is therefore required to overcome these distractions from their home environment as far as virtual learning is concerned.

The current study showed that the majority of nursing students experienced financial challenges mainly associated with purchasing internet data bundles, consistent with earlier studies which uncovered some variables such as financial difficulties and technological obstacles as some challenges faced by nursing students [36,37]. Financial wellbeing among students is correlated with academic success and graduation rates [38,39]. These findings emphasize the significance of addressing financial challenges faced by nursing students to ensure their overall wellbeing and academic success. This can be realized by providing appropriate support and resources to mitigate financial burdens such as scholarships, educational institutions can contribute to improving the success and retention rates of nursing students.

Consistent with Li, et al. [40], Mokhtari, et al. [41]and Conrad [42], the study also sheds light on some limitations of the virtual medium. Participants reported that some students became less engaged and motivated, potentially leading to absenteeism and academic dishonesty. While online learning offers flexibility, it also requires students to be self-disciplined and committed to their studies. Successful participation of students in the virtual learning platform requires the proactiveness, self-motivation and discipline of students to take control of their learning process. In addition, the lack of stringent supervision in the virtual medium was cited as a factor contributing to academic dishonesty, such as copying quizzes and assignments. Yeung and Yau [43]acknowledge the challenges of ensuring academic integrity in online assessments and highlights the need for implementing effective strategies to deter cheating.

Finally, the study revealed that not all students were equally prepared for the transition to the virtual medium. Some participants struggled with studying alone at home and felt unready for the shift. Similar findings have been reported by Singh, et al. [44] and Zhao and Xue [45]where they reported on the unpreparedness of both students and teachers in migration to online studies due to lack of support and poor orientation. This highlights the importance of providing adequate support and resources to help students adapt to online learning effectively. Literature further emphasizes the significance of orientation programs and support services to assist students in navigating the virtual learning environment [44,45].

## 5. Conclusion

The study’s findings have shown that using virtual learning environments during the COVID-19 pandemic is related to a number of expectations, experiences, and challenges. The study emphasized a variety of elements that have an impact on nursing students’ virtual learning such as the acquisition of ICT knowledge and skills, continuous internet connectivity, uninterrupted electricity supply, improvement in academic grades, and pre-training. These interconnected elements presented by the participants demonstrate that everyone—the individual, their family, peers, institutions, and policymakers—has a part to play in enhancing the virtual education environment so that students are empowered in their learning process to achieve targeted learning outcomes.

## 6. Limitations of study

- This is a qualitative study so the findings cannot be generalized to all populations. However, a detailed description of the study setting has been provided to enhance transferability.
- The study involved participants from only one educational institution in Ghana that potentially has better infrastructure and services to support virtual learning as compared to other institutions within the country. Thus, the experiences and perspectives may be different from students in educational institutions with less developed infrastructure to support virtual learning.

## Supporting information

S1 File Interview guide. (DOCX)

S2 File Interview transcript. (DOCX)
