# Supplementary material for: Expectations, experiences and challenges of nursing students using the virtual learning medium during the COVID-19 pandemic: A descriptive phenomenological study
Source: PLoS One. 2024 Mar 8;19(3):e0299967. doi: 10.1371/journal.pone.0299967 (PMC10923445; doi:10.1371/journal.pone.0299967)
Supplement: S1 File — (DOCX) [file pone.0299967.s001.docx]

INTERVIEW GUIDE

1. Please tell me about yourself

- Age
- Gender
- Program
- Year of study
- Religious background

1. Please share with me how the covid-19 pandemic impacted your learning?
2. Explain to me your understanding of the virtual medium of education?
3. Please share with me the types of virtual learning platforms offered to you by your university.
4. Please share with me your virtual learning experiences?
5. What were your experiences about the differences between the traditional face-to-face and the virtual medium?
6. How beneficial is the virtual medium of education to you?
7. What expectations did you have about the virtual medium of education?
8. Kindly describe the extent to which these expectations have been met?
9. What did you expect with your grades using the virtual medium?
10. What did you gain from the virtual medium of education?
11. Moving into the future, what do you see as the prospect of the virtual medium of education, especially in Ghana?
12. Please share with me the challenges encountered using the virtual medium.
13. Kindly describe the nature of the difficulties you encountered using the virtual medium.
14. What were the factors that facilitated your virtual learning?
15. What structures were put in place by your university to assist you in your virtual learning?
16. Kindly describe the support offered to you by your university to help in your virtual learning
17. What are your virtual learning recommendations?
18. Is there anything else you would like to comment on about the virtual medium of education that we have not spoken about or discussed today?
