# Supplementary material for: Expectations, experiences and challenges of nursing students using the virtual learning medium during the COVID-19 pandemic: A descriptive phenomenological study
Source: PLoS One. 2024 Mar 8;19(3):e0299967. doi: 10.1371/journal.pone.0299967 (PMC10923445; doi:10.1371/journal.pone.0299967)
Supplement: S2 File — (DOCX) [file pone.0299967.s002.docx]

**RESEARCH INTERVIEW TRANSCRIPT**

**IN-DEPTH INTERVIEW WITH NS001**

R: please how old are you?

NS001: I am **24 years old**

R: please tell me your educational level?

NS001: I am level 300.

Gender: F

R: please, what program do offer?

NS001: BSc. Nursing

R: please what is your religious background?

NS001: I attend the church of Pentecost

**R: Please share with me how the covid-19 pandemic impacted your learning?**

**NS001**: When the covid-19 came, has impacted my learning in both positive and negative ways. I am not an independent learner. But with the covid-19 emergence and the use of the virtual medium, I had to make independent learning and discipline part of me. And this helped me. Negatively, I can say that the data bundle was too costly for me. Besides, my location was not good enough as I had network challenges. And this affected my virtual learning.

**R: What is your understanding of the virtual medium of education?**

**NS001**: The virtual medium of education to me is a means or way of acquiring knowledge via electronic devices using the internet. I see it as having education outside of the physical campus classroom. Having education at your convenient place.

**R: please share with me the types of virtual learning platforms offered to you by your university?**

**NS001**: Our school adopted zoom and google classroom. Before the covid, the university uses the V-class. But we commonly used the zoom platform for lectures during the covid-19 pandemic. I prefer it to others

**R: tell me why you prefer the zoom app?**

**NS001**: The zoom app is a virtual platform or software that enables students to partake in learning in real-time. It has helped me a lot. I am a shy person and so I do not like contributing to the physical class due to the physical presence of colleagues. But with zoom, I could actively partake in the virtual class by answering and asking questions. This is because I am with my computer and myself and no one else. However, my problem with zoom is that, when I missed a zoom lecture, I have to make up for it personally.

**R: What are your virtual learning experiences?**

**NS001**: I had a personal computer, but it got faulty. And so when the covid-19 pandemic emerged and we have to go virtual, what I did was buy a phone to be able to take part in the virtual medium of education. I am not an independent learner, to be honest with you, but with the covid-19 pandemic emergence and the subsequent use of the virtual medium, I developed an attitude of learning independently of my friends as well as my lecturers. It became a habit for me to go on to the internet and search for information for myself for purposes of education.

**R: what are the differences between the traditional face-to-face and the virtual medium that you experienced?**

**NS001**: With the traditional face-to-face the lecturer is physically present and so students pay attention in class. But with the virtual medium, I can attend lectures from a distance. The lecturer to some extent may not have absolute control of the virtual classes in terms of attendance. For instance, there are situations where I did not log on to zoom lectures and even with those that did, could not complete the virtual class. I was distracted whilst at home using the virtual medium. For example, during zoom lectures, I may be receiving calls, WhatsApp messages, or even playing music among others.

**R: how beneficial is the virtual medium of education to you?**

**NS001**: The virtual medium has enhanced my skills in ICT. Even though I had a little knowledge about how to use a computer and smartphones, I did not use them consistently and so it became as if I had not had any knowledge of the use of the ICT tools. But with covid-19 emergence and the use of the virtual medium, my technical skills were much more improved.

**R: what expectations did you have about the virtual medium of education?**

**NS001**: ok. I was expecting the government or the school management to support us adequately during these trying times of covid-19 pandemic so far the virtual learning is concerned. For example, support like providing us with effective internet(WIFI) at no cost, stable electricity to help us have an effective virtual medium education free of distractions.

**R: To what extent did you meet your expectations?**

**NS001**: Alright, if I am to rate on a scale of 0-100 percent, I will say 50% expectations were met. This is because some data bundle was provided to us even though inadequate for the virtual learning. Also, we had access to the ICT laboratory with some assistance from the IT experts which helped us have effective virtual learning.

**R: What did you expect with your grades using the virtual medium?**

**NS001**: Despite the sudden shift from the traditional face-to-face to the virtual medium, my grades were very good. My grades were better compared with the traditional face-to-face medium.

**R: What did you gain from the virtual medium of education?**

**NS001**: I will say that I was able to acquire some skills in ICT. I am now able to use these ICT tools like laptops and the virtual medium software with little difficulty.

**R: moving into the future, what do you see as the prospect of the virtual medium of education, especially in Ghana?**

**NS001**: Alright, I will say that, if Ghana as a nation is committed to ensuring that students use ICT tools in every educational institution, then the virtual medium is here to stay. But this can only be possible if the government gives adequate support to this agenda.

**R: Could you please share with me the challenges encountered using the virtual medium?**

**NS001**: Alright, to me I had some challenges with the virtual medium. One was poor internet connectivity and as I said earlier, the cost of the data bundle was also a challenge. My environment was full of distractions. For instance, you may be in your room having a lecture on zoom and suddenly someone calls you out for a discussion and that distracts you a lot.

R: What were the factors that facilitated your virtual learning?

NS001: For me what helped me in my virtual learning was because I had a very good android phone with powerful specifications in speed and capacity that made me enjoy the virtual medium of education. Some of my colleagues had challenges that did permit them to use the virtual medium.

R: what structures were put in place by your university to assist you in your virtual learning?

NS001: The ICT lab was made available 24hours for us. The university ICT lab was equipped to help students with or without laptops and android phones to have effective virtual learning.

R: What are your virtual learning recommendations?

NS001: I will recommend to the university and government to support all educational institutions with adequate internet connectivity. Also, an upgrade or construction of a state of the art ICT laboratories centers for higher education institutions like ours will be appropriate to support the virtual medium of education going forward. With this, I think many students will appreciate and embrace the virtual medium of education.

R: please add anything concerning the virtual medium of education during the covid-19 pandemic that we couldn’t talk about during this interaction?

NS001: I think all the information I wished to give concerning the virtual medium of education during the covid-19 pandemic is exhausted.

R: thank you for your time, God bless you.

NS001: thank you too.

**RESEARCH INTERVIEW TRANSCRIPT**

**IN-DEPTH INTERVIEW WITH NS002**

R: please how old are you?

NS002: I am **32 years old**

R: please tell me your educational level?

NS002: Mphil Nursing, level 600

Gender: M

R: please what is your religious background?

NS002: I am catholic

**R: Please share with me how the covid-19 pandemic impacted your learning?**

**NS002**: The covid-19 pandemic that befell us in 2019 came with a lot of challenges and a lot of opportunities opportunities in a sense that you are able to combine your number work schedule and be able to school at the same time I like in the past where you will move from my location to another to be able to have your education so that's one positive impact of coverage on my learning looking at it negatively you can see that the internet connectivity the platform created an opportunity to have lectures Wiles at our convenience places also what are the negative impacts was with the internet connectivity for example if you find yourself working in a very remote area you are able you are unable to have good internet connectivity and that can go a long way to affect you negatively in terms of your lead another negative impact of the visual medium or the covid-19 is that there was high cost of data as we all know data in Ghana is very expensive unlike in other parts of the advance countries you can have those in Europe always advocating for tacos to make internet access very cheap but here in Ghana it is cheaper to make phone calls that because you have to spend a lot of birthday even though the virtual video provides you with the convenience of not travelling to attend lectures I think this is how the government in poverty has impacted by Lady

**R: explain to me your understanding of the virtual medium of education?**

**NS002**: With the virtual video medium of education I see it has having you're learning via the internet's using electronic devices, for example, you can have your lectures via WhatsApp Zoo Google beats and so on so you will have the lecturer at one end of the medium delivering lectures and whatever you are as a student you connect 88 parts at the other end of the video using the internet.

**R: please share with me the types of virtual learning platforms offered to you by your university?**

**NS002**: At KNUST, the virtual platform called V-class was the university platform provided to us to access using your ID and password. we also had lecturers who delivered their lectures using the zoom app, Google meet, and sometimes Whatapp.

**R: Which of these virtual platforms did you prefer?**

**NS002:** My preferred platform was the zoom app because it is easy to install. I could use it with little difficulty. it was the most commonly used virtual learning platform during the covid period. However, you will always need to have the internet to be able to hook on to it.

**R:: What are your virtual learning experiences?**

**NS002**: With my experiences, I can say that it was fun as it offered me the opportunity to have my lectures in the comfort of my home and not necessarily travel from home to campus for lectures. For instance, I work at tamale in the northern part of Ghana so if not with the visual medium it means I needed to travel from Tamale to Kumasi to have lectures. so with this virtual, it has saved me time and resources from traveling up and down. It also improves my internet usage and my ability to manipulate the virtual medium software and other electronic devices.

**R: What are your experiences about the differences between the traditional face-to-face and the virtual medium?**

**NS002**: As I indicated earlier with the virtual medium, I could sit in the comfort of your home with internet enable connectivity and be able to take part in the virtual class but with a traditional face-to-face you will have to move to the class physically to have lectures. The virtual medium is very flexible as compared to the traditional face-to-face.

**R: how beneficial is the virtual medium of education to you?**

**NS002**: It makes learning easier for me. I did not need to travel to attend lectures with its associated cost. I could even participate in the virtual lecture whiles in the washroom.

**R: what expectations did you have about the virtual medium of education?**

**NS002**: I expected that with the virtual medium the traditional mode of delivering lectures and its challenges will be a thing of the past. So I expected the virtual medium could be used to assess students without compromising the system.

**R: please to what extent did you meet your expectations?**

**NS002:** Yes, I can say to some extent some expectations have been met because I was able to acquire the necessary skills that came with the virtual medium. However, the quality of delivery and assessing students were not met at all.

**R: What did you expect with your grades using the virtual medium?**

**NS002:** My grades obtained using the virtual medium were not different from using the traditional face-to-face medium. I did not see any significant difference in my performance using the visual medium, even though it came it some challenges.

**R: What did you gain from the virtual medium of education?**

**NS002**: As I indicated earlier, my research and ICT skills have improved tremendously since using the virtual medium.

**R: moving into the future, what do you see as the prospect for the virtual medium of education, especially in Ghana?**

**NS002**: I think the virtual medium of education is here to stay whether we like it or yes. Its advantages of convenience, flexibility and cost-effectiveness make it appropriate for both students and instructors. I think this is only possible if the government of Ghana is committed to its digital agenda. So I do recommend struggling that the eventual medium should be maintained even after the covid-19 pandemic.

**R: Could you please share with me the challenges you encountered using the virtual medium?**

**NS002**: It was very costly for me to get data. I also faced tough difficulties acquiring the appropriate electronic devices such as laptops, smartphones, and modems because of my low socioeconomic background. Besides some of the lecturers could not manipulate these virtual mediums as they were new and affected us as students. Let me also add that, the issue of unstable internet connectivity also affected me.

**R: what was the nature of the difficulties you faced using the virtual medium?**

**NS002**: Let's take the example of the cost of data. For instance, with 20ghs data was not insufficient for me to join a virtual class for 3hrs. This incident happened to me during the first few days of virtual learning.

R: What were the factors that facilitated your virtual learning?

NS002: A friend of mine supported me with her laptop. Also, the school provided us with some data to assist us in our virtual learning.

R: what structures were put in place by your university to assist you in your virtual learning?

NS002: Data monthly was offered to us even before the covid-19 pandemic but I must say with the covid-19 emergence and the subsequent adoption of the visual medium that data package became woefully inadequate. Also, Wi-Fi was provided to us on-campus which brought some relief to us on campus.

R: Could you please describe the support offered by the university to help you in your virtual learning during the covid-19 pandemic?

NS002: For me, I think the Wifi was one very important support so far as the virtual medium of education is concerned. The creation and adoption of the various virtual media made our virtual learning possible. They were also resourced persons available for students to contact in case of difficulty with the virtual medium.

R: What are your virtual learning recommendations?

NS002: I will recommend that the data component of the virtual medium should be borne by the university. they should partner with telecommunication networks such as MTN or Vodafone to make data affordable for students. The university can also procure electronic devices specifically for virtual learning activities rather than allowing individual students to use their phones and laptops. Training should also be given to both faculty and students to improve the experience and effectiveness of the virtual medium.

R: Is there anything else you will like to comment on about the virtual medium of education that we have not spoken about?

NS002: I think all that I wanted to talk about have being mentioned

R: thank you for your time, God bless you.

NS002: thank you too.

**RESEARCH INTERVIEW TRANSCRIPT**

**IN-DEPTH INTERVIEW WITH NS003**

R: please how old are you?

NS003: I am **27 years old**

NS003: BSc Emergency Nursing

R: please tell me your level?

NS003: I am in level 400

Gender: F

R: please what is your religious background?

NS003: I am a Jehovah's Witness

**R: Please share with me how the covid-19 pandemic impacted your learning?**

**NS003**: Okay, first of all, the covid-19 pandemic had both positive and negative experiences for me. Let me start with the s positive experiences. when the Covid-19 pandemic came, we started using the virtual medium by moving from the traditional face-to-face which enabled me to study regardless of my geographical location. For example, I could be studying while in a car or the kitchen. We were supported with data to enable us to have an effective virtual learning experience. I had to acquire a new phone to be able to partake in the virtual classes. One impact I experienced was the distraction with the virtual whilst at home. My family and other activities did allow me to have a successful virtual medium education at home. Most students copied assignments and quizzes and past them on as their own whilst using the virtual medium. To me, the virtual medium could control or check students on issues of cheating and examination malpractices as students got away with it. So in the area of assessment, the virtual medium did not help us at all. It rather created laziness and indiscipline among students.

**R: explain to me your understanding of the virtual medium of education?**

**NS003**: The virtual medium simply means using the internet to gain access to two educational materials and lectures without being in a physical classroom. Students engage in learning from a distance but are still able to interact with their lecturers.

**R: please share with me the types of virtual learning platforms offered to you by your university?**

**NS003**: Ok, we had some virtual platforms which included the virtual class, the zoom app the Google class, and WhatsApp. I prefer zoom because it was easy to use and I could get instant feedback during a lecture using it.

**R: please share with me your virtual learning experiences**

**NS003**: My experiences are both good and bad. Money was readily available for me to purchase data for my virtual learning. The virtual was very easy for me to use as I could learn from anywhere provided I had an internet connection. But I must say that the virtual medium brought about some laziness and a bad attitude toward learning. I think assessing students using this virtual medium in their current form is appropriate because of malpractices.

**R:: please could you share with me your experiences about the differences between the traditional face-to-face and the virtual medium?**

**NS003:** Ok with the virtual medium I could have lectures anywhere regardless of my physical presence in the classroom of the campus, unlike the traditional face-to-face that demands your physical presence in class. I could not succeed in using a virtual class without internet connectivity. But with traditional face-to-face, no internet connectivity is needed to have a class.

**R: how beneficial is the virtual medium of education to you?**

**NS003**: I was able to manage my time very well whilst using the virtual medium. For instance, there was a day I needed to attend a friend's funeral and also have a zoom lecture on the same day and time. So I had to audio record the lecture whilst at the funeral grounds, and this enables me to succeed with the two events concerning managing my time. personally, it was the most flexible learning medium I ever experienced. Besides this virtual medium also improved my communication skills and boosted my confidence level in participating in virtual classes.

**R: what expectations did you have about the virtual medium of education?**

**NS003**: Ok my expectations were not that high as I expected to have the same experience I had using the traditional face-to-face medium. But this was even better than the traditional face-to-face medium because I could record and listen to two lectures at a later date with the virtual medium.

**R: please kindly describe the extent to which these expectations have been met?**

**NS003**: Ok, let me say that my expectations to a large extent were partially met because I got distracted very easily while at home so sometimes there are things I am supposed to understand but I don't do it as home activities do not allow me to concentrate when using the virtual medium.

**R: What did you expect with your grades using the virtual medium?**

**NS003**: I expected my grades to be good and even better than using the traditional face-to-face medium because I had the opportunities to learn on my own. For instance, any time we are given a question to solve, because it's virtual, going online becomes the easiest way to find your answers, unlike face-to-face where phones are not allowed in class. So, I had good grades using the virtual medium.

**R: What did you gain from the virtual medium of education?**

**NS003**: I was able to learn some skills in ICT. Because in this part of the world everything is changing so adequate knowledge in ICT will help me so much. For instance, right now almost all hospitals are incorporating ICT into their operations. So to keep up with current trends in this global village, one needs to have adequate knowledge about ICT.

**R: moving into the future, what do you see as the prospect of the virtual medium of education, especially in Ghana?**

**NS003**: I think that the virtual medium has great potential for both teachers and students in Ghana. This is because of how flexible and convenient it is. And I believe that students will be more than willing to embrace it provided policies are put in place to make it more reliable.

**R: Could you please share with me the challenges encountered using the virtual medium?**

**NS003**: I encountered a lot of challenges. Economically I could not purchase data for my virtual learning activities during the covid-19 pandemic. In fact, at some point, it looks like I was demanding too much money for data every day from my parents. Meanwhile, I am not the only child of my parents and so that affected the family finances. Erratic power supply affected my virtual learning so much that any time there was a power outage my virtual learning get truncated. Besides, some features of the virtual medium software were difficult to learn within a short period.

**R: Could you please describe the nature of the difficulties you encountered using the virtual medium?**

**NS003**: Ok the nature of the difficulties I faced were the economic issues with internet data bundle purchase. I had difficulty accessing the virtual medium due to poor internet connectivity and sometimes when you are home you have difficulty paying attention due to distractions from your environment which could be either relatives or noise from vehicular movement or animals.

R: What were the factors that facilitated your virtual learning?

NS003: I had good internet Wi-Fi at home. My dad had Wi-Fi that supported me so much with my virtual learning during the early part of the pandemic as we were home. initially, my dad did not allow me to log on but when the covid came and we virtual I was permitted to use the Wi-Fi and that helped me a lot. Besides my dad realized the importance of education and got me a new phone that enabled me to participate in virtual learning.

R: what structures were put in place by your university to assist you in your virtual learning?

NS003: The university provided us with 2.5 gig of data every month but with the covid-19 emergence the data bundle was increased to 5 gigs and that helped us. besides some virtual platforms were provided to us that could be accessed even offline and that took some burden off us financially.

R: Could you please describe the support offered to you by your university to help in your virtual learning

NS003: The school gave us some internet data bundle. ICT experts were readily available to assist us with any challenges we may encounter with our virtual learning activities

R: What are your virtual learning recommendations?

NS003: Yes so I recommend that the government can help those, especially in the remote areas with Internet data packages. Also, ICT gadgets like laptops could be provided to students in deprived areas to help them have effective virtual learning. Besides, I recommend that university management should get additional power plants in addition to the National Grid to take care of power outages. I believe this will help students have an uninterrupted virtual learning experience.

R: Is there anything else you would like to comment on about the virtual medium of education that we have not spoken about or discussed today?

NS003: Ok I will say in all the virtual medium is good. The government should try its very best to help incorporate ICT into various educational institutions.

R: Thank you so much for your time and the information you have provided today

NS003: Thanks for having me

**RESEARCH INTERVIEW TRANSCRIPT**

**IN-DEPTH INTERVIEW WITH NS004**

R: please how old are you?

NS004: I am **25 years old**

R: What program do you offer?

NS004: BSc Midwifery

R: please tell me your level?

NS004: level 300

Gender: F

R: please what is your religious background?

NS004: I am an Anglican

**R: Please share with me how the covid-19 pandemic impacted your learning?**

**NS004:** My experience with a covid-19 pandemic, in the beginning, was not easy. Because we have to leave lectures and go stay at home. But when we started with the virtual medium things became easier. I could attend to recorded audios in my own free time and could make my notes in addition to the lecture notes. And so I think this was a bit positive for me, but there were some difficulties too. For instance, when lectures are going on, for example, using the zoom app sometimes you might be so busy that it’s difficult to join the virtual class whilst at home.

**R: What is your understanding of the virtual medium of education?**

**NS004**: I understand it as a means of continuing our academic exercises or duties using the internet.

**R: please share with me the types of virtual learning platforms offered to you by your university?**

**NS004**: Ok I can talk about the vclass, zoom, google class, and WhatsApp. With vclass, information, such as assignments and quizzes are posted there for us to download. However, with zoom, it’s like a video application where you are given a passcode and ID to access the lectures. So we were offered both synchronous and asynchronous virtual platforms.

**R: please could you share with me your virtual learning experiences?**

**NS004**: As I already said earlier, it wasn't easy at the beginning. Most of the time, my poor network did allow me to have a good virtual medium education experience. I could join a virtual class and suddenly, the network gets jammed and I lose out on everything, especially with the zoom. Even though the university provided support with data it wasn't insufficient for the virtual medium. So we still have to spend some money to purchase data for the virtual medium and this was a bit stressful for me economically.

**R: please could you share with me your experiences about the differences between the traditional face to face and the virtual medium**

**NS004**: yes, there are so many differences. Let me start with the student presence. When it’s time to have a lecture using the traditional face-to-face there is a hundred percent chance of you being present but with a virtual, it is a fifty percent chance of being available. The traditional face-to-face interaction with lecturers is interpersonal and physical. But with the physical, the interaction takes place from a distance. Sometimes because of the cost of data we wish the virtual classes were made short to save our data. So because of that, it becomes difficult to contribute meaningfully to the virtual class. Also with the virtual medium, feedback with assignments was more swift than the traditional face-to-face.

**R: how beneficial is the virtual medium of education to you?**

**NS004**: It is beneficial to me in the sense that I could complete the semester on time and did other businesses to gain some money. I could fall on the audio-recorded lectures any time I missed lectures. We had feedback with the assignment earlier using the virtual medium than the traditional face-to-face.

**R: what expectations did you have about the virtual medium of education?**

**NS004**: I was expecting that the university will bear the cost of data for us. Unfortunately, the data package provided to us was not sufficient for our virtual learning. I also expected the lecturers to reach out to us on our opinion about the virtual platforms to improve upon them. I also expected a follow-up by lecturers on how the assignment was done using the virtual medium. You know that covid-19 came with a lot of surprises and financial constraints and we know these electronic gadgets can be faulty at any time. So I thought the government or the university could assist us with these electronic gadgets to enable us to take part in virtual learning. Ghana suffers erratic power supply commonly called dumsor, so I was expecting that additional power plants could be provided and deadlines of assignments usually extended because of the power problem but all these things did not happen it affected me so much.

**R: Could you please describe the extent to which these expectations have been met?**

**NS004**: Ok I think there was a day a lecturer gave an assignment and before we knew the deadline for submission was due, by then most of the students could not submit on time so we contacted her and she extended the deadline and we were able to submit the assignment so I think she was the one by her kindness who met my expectations. Besides, my ability to access audio recordings using virtual applications was awesome for me.

**R: What did you expect with your grades using the virtual medium?**

**NS004**: With my grades, although there were difficulties with the virtual medium that I spoke about earlier, I expected my grades to improve using the virtual medium because I was able to assimilate a lot of things about the lectures given. I put in so much effort because the virtual medium, I believe it’s meant for independent and disciplined learners. And so I was able to understand and search for other information which could be very beneficial to my learning. The period given for assignment submission was adequate for most of the courses and I think that made my results better.

**R: What did you gain from the virtual medium of education?**

**NS004**: I gained some computer skills and how to navigate important websites for educational purposes to enhance my education. I was able to do more than one thing at the same time whilst using the virtual medium.

**R: moving into the future, what do you see as the prospect of the virtual medium of education, especially in Ghana?**

**NS004**: I think virtual learning is going to be sustained because if you observe, you will realize that distance is one factor that impedes education. And so with the virtual medium distance is no barrier to nursing education. Besides, we are now living in the time of the digital age and things evolve so institutions like banks and health facilities across the country are going digital. So I think it can be sustained because teaching and learning will be convenient and flexible and cheap for all students. So I see the virtual medium as having a better future in Ghana. However, the government and the University management must have to intervene with the issues of the internet connectivity, power supply data bundle, and access to ICT gadgets for students.

**R: Could you please share with me the challenges encountered using the virtual medium?**

**NS004**: I encountered several challenges using the virtual medium. First is the cost of data. Because of how expensive data is I could not connect to the virtual medium on some days. Many students absented themselves from the virtual class because of a lack of monitoring and control with the virtual medium. Poor network resulting in bad internet connectivity was I serious issue I was confronted with. One challenge I also faced was the inability to see other top management members of the school who will usually come to talk to us from time to time and motivate us to learn.

**R: Could you please describe the nature of the difficulties you encountered using the virtual medium?**

**NS004**: Ok as part of my challenges I made mention of the internet and poor network. With the poor internet connectivity I remember there was a time we had a zoom class and all of a sudden the network failed and before I could reconnect, I was informed the lecture had ended. I felt so sad that day.

R: what factors facilitated your virtual learning?

NS004: My parents bought me a router following the challenges I had with my internet connectivity so with a router I could enjoy my Virtual class without any difficulty or distraction. So I think this is facilitated by virtual learning.

R: what structures were put in place by your university to assist you in your virtual learning?

NS004: The University provided the virtual class alongside other virtual applications like zoom for students to use for their virtual education. This allowed us to download lecture notes and also submit assignments. The university also provided us with free Wi-Fi which aided our virtual learning.

R: Could you please describe the support offered by the university to help you in your virtual learning

NS004: yes, one thing the university did that I was really happy with was that during the covid period resources persons in ICT were assigned to those lecturers who had difficulties using the virtual medium to assist them to have successful virtual teaching by providing them with the appropriate computers and stable internet connectivity to enable them to engage with students on the virtual medium. The University management in collaboration with Vodafone Ghana offered a data package to students for virtual learning.

R: What are your virtual learning recommendations?

NS004: To me, for this virtual medium to be effective they should be an unlimited supply of internet to both students and faculty. I also recommend some training workshops organized for both students and faculty on the use of virtual platforms. Government should provide support in the provision of ICT Laboratories to help students have an effective virtual education. Government can also train ICT expects particularly software developers to develop virtual medium software that is cheaper and easy to use within the Ghanaian context to help in virtual learning.

R: please add anything concerning the virtual medium of education during the covid-19 pandemic that we couldn’t talk about during this interaction?

NS004: Ok one thing I would like to say is we all came to the university with different socioeconomic and geographical backgrounds Students from Paul or deprived areas should be assisted with the necessary assistance to help their benefits from the virtual medium of education

R: thank you for your time, God bless you.

NS004: thank you too.

**RESEARCH INTERVIEW TRANSCRIPT**

**IN-DEPTH INTERVIEW WITH NS005**

R: please how old are you?

NS005: I am **26 years old**

R: please, what program do you offer?

NS005: I offer BSc. Midwifery

R: please tell me your educational level?

NS005: I am in level 300.

Gender: F

R: please what is your religious background?

NS005: I attend the church of Pentecost

**R: Please share with me how the covid-19 pandemic impacted your learning?**

**NS005**: Ok, so somewhere around last year in March we received information from the president that all universities should be on lockdown and students send home by then I was preparing for a quiz in surgical nursing. So although I was happy, I also felt that this pandemic will distract our academic calendar. One of my concerns was the fact that our stay on campus was going to be prolonged. A week after we left campus and the physical classes were suspended, a message was sent to us via WhatsApp that the traditional face-to-face classes were going to be moved to a virtual medium. For me, it was a piece of welcoming news. However because it was a new thing to us and how suddenly it was adopted, it affected my learning greatly. I am not that good at manipulating ICT tools. Besides we were not taken through any training on virtual platforms like Zoom before their adoption. So it made learning difficult for me within the first few months of the lockdown. However as time went on, and with the assistance of colleagues on the use of the virtual platform live zoom as well as my trial and error attitude, I was able to find my way around the virtual platforms and have a somewhat successful virtual medium education during the covid-19 pandemic. So I will say it was uncertainties at the Beginning characterized by frustration and anxieties to adaptation and learning new skills using the virtual medium.

**R: explain to me your understanding of the virtual medium of education?**

**NS005**: Ok as I experienced it, the medium I will say is a medium where teaching and learning take place over the internet using ICT tools like laptops and smartphones. As students, we could get instant feedback when using the zoom app. However, with the vclass, we could not get feedback instantly.

**R: please share with me the types of virtual learning platforms offered to you by your university?**

**NS005**: Ok for my University, they adopted the vclass, Zoom app, WhatsApp platform, Google class, and Google meet.

**R: please share with me your virtual learning experiences**

**NS005**: So with my virtual learning experiences, some are funny and others are a bit worrying and all that, but in the end, I learned something. First, let me start with the distractions in the house. It wasn't easy at all. This is because I am the only female in the house and activities like cooking washing and cleaning did not permit me to have satisfactory virtual learning. So the home responsibilities and having lectures alongside were very tedious for me but I manage with it like that. The next thing I want to talk about is the cause of the internet data. It was a challenge for me because as a dependent I did not have any source of income except my parents. Mind you, I am not the only child of my parents so demanding extra cash for a data bundle was draining the family coffers. But my dad was able and committed to helping me in my virtual learning. Besides electronic gadgets like the smartphone, modems and laptops do fail us sometimes and that was an issue of concern to me. For instance, there was a day I went to the farm with my mum in the process of farming I realized that I was to have a lecture in the next 30 minutes. My mum then asks me to go and partake in the virtual class. I got home and as I log on to zoom, my battery suddenly ran low on power, and before I knew it the lecturer had finished the lecture. So that incident affected my virtual learning that day.

**R: please share with me your experiences about the differences between the traditional face to face and the virtual medium**

**NS005**: Ok, so the traditional face-to-face is where a teacher is physically present with students in a physical class. But with the virtual class teaching and learning is conducted over the internet using electronic devices like laptop model and smartphones. With the virtual medium teaching and learning takes place regardless of geographical location. Usually, data is needed to get access to a virtual class. Student concentration and class participation can be monitored using the traditional face-to-face as compared with a visual medium. For example with the zoom app, students don't like to have their video switched on during virtual lectures which makes it difficult to know who is present or not. With the virtual medium, you cannot even know who is following your lectures. So students are more actively involved in the traditional face-to-face medium than the virtual medium. Absenteeism is an order of the day with the virtual medium than the traditional face-to-face.

**R: how beneficial is the virtual medium of education to you?**

**NS005:** To me, the virtual medium had a lot of negative things about it but I think the negative issues came in because of how suddenly it was adopted and the fact it was a new thing to us. So, my everyday try and error made me learn new skills in ICT during the learning process using the virtual medium. Initially, I did not like playing with these electronic devices like the laptop among others but with this virtual medium I learned some ICT skills that helped me to manipulate these electronic gadgets successfully

**R: what expectations did you have about the virtual medium of education?**

**NS005**: My first expectation was that I thought the virtual medium of education could be conducted for free. So, I realized that there was some cost component to it, I was expecting that students will be supported with adequate data to participate. Besides I was expecting to be able to use the zoom app with ease. But its use came with some difficulties. I expected the assessment of students using the virtual medium to be similar if not the same as the traditional face-to-face. I also thought the virtual medium was adopted permanently but as the covid-19 subsided coupled with the preventive protocols in place, my department reversed to the traditional face-to-face I was disappointed with the decision of reverting to the traditional face-to-face because can you imagine that our licensing exams are written online. So I thought it was an opportunity for us to be rehearsing only to be disappointed. Finally, I want to say that the virtual medium could support clinical skills tutorials and affected us.

**R: please kindly describe the extent to which these expectations have been met?**

**NS005**: I was able to acquire some technical skills in the use of electronic devices with the virtual medium as an expectation.

**R: What did you expect with your grades using the virtual medium?**

**NS005**: Because of the challenges I encountered my grades were not good. Comparing my performance with the virtual medium to the traditional face-to-face I must say it was very abysmal.

**R: What did you gain from the virtual medium of education?**

**NS005:** Even though I have some challenges at the initial stage I learned something new with the use of electronic gadgets such as laptops and smartphones I was also able to manage my time very efficiently because the virtual medium is very flexible and so I could multitask during the lockdown whilst using the virtual medium.

**R: moving into the future, what do you see as the prospect of the virtual medium of education, especially in Ghana?**

**NS005**: The future of virtual medium begins with how the government will involve itself in its digital technology business. As we speak now I am aware that almost all hospitals in Ghana are using electronic health records software in their institutions. So to keep up with this digital age government in collaboration with the university should inculcate into the curricula the virtual medium of education. If this happens, I can tell you that the virtual medium will have a bright future in Ghana. Already the NMC Ghana has set the tone for a digital take off in the nursing profession, so I think the virtual medium is here to stay

**R: Could you please share with me the challenges encountered using the virtual medium?**

**NS005**: One of the challenges I encountered was the network for internet connectivity. Sometimes I have to move from my house to a different area to access a stable network for my internet connectivity. Getting a data bundle for the virtual medium was very difficult for me because of the cost of the data. This was because I did not have money for data. Even though we were given some data but I was insufficient for the virtual learning. Distractions from home are also affected by the virtual learning experience.

**R: Could you please describe the nature of the difficulties you faced using the virtual medium?**

**NS005**: As mentioned earlier, the poor network was my biggest challenge or difficulty. Let me tell you why it is. On this fateful day, I left home in search of a good network for my virtual class. I finally found a place with a good network and in the process of the virtual learning, the rain came the whole and so my virtual learning did not do not happen that day. I will never forget that day.

R: What were the factors that facilitated your virtual learning?

NS005: My daddy's commitment to education made him appreciate the virtual medium of education and so he provided me with the needed electronic gadgets to help me in my virtual learning. Also, the sacrifices made by the lecturers contributed to successful virtual learning for me. finally, that self-motivation within us as students to engage in virtual learning also facilitated our learning using the virtual medium.

R: what structures were put in place by your university to assist you in your virtual learning?

NS005: Yeah okay so the structures put in place have to do with the use of the vclass, the zoom app, and the WhatsApp platform.

R: What support was offered to you during the virtual medium?

NS005: Ok so the support the University offered to us came in a form of a data package monthly even though at some point it became inadequate to enable us to have a virtual lecture. But it was better than none.

R: What are your virtual learning recommendations?

NS005: With my recommendations, the government should partner the universities to drive the digital technology agenda in the country. Pre-training workshops should be organized for the students after admission into these educational institutions that use these virtual mediums of education. I also recommend that ICT training and teaching should be taught starting at the early stages of education say the basic schools. This will make students familiarise themselves with these electronic gadgets. I think the government needs to invest heavily in the ICT industry if they want to have the virtual medium of education being part of our curricula.

R: please add anything concerning the virtual medium of education during the covid-19 pandemic that we couldn’t talk about during this interaction?

NS005: Ok basically we have talked about almost everything I guess

R: thank you for your time, God bless you.

NS005: thank you too.

**RESEARCH INTERVIEW TRANSCRIPT**

**IN-DEPTH INTERVIEW WITH NS006**

R: please how old are you?

NS006: I am **22 years old**

R: please, what program do you study?

NS006: BSc Nursing

R: please tell me your level?

NS006: I am in level 300.

Gender: M

R: please what is your religious background?

NS006: I am a Methodist

**R: Please share with me how the covid-19 pandemic impacted your learning?**

**NS006**: The covid-19 pandemic change the way teaching and learning were done. The impact was both favorable and unfavorable to me. With the unfavorable impact, learning from a distance for me was full of distractions from family members, noise, etc. Most of us did not concentrate in the virtual classes as students were engaged in other activities such as WhatsApp chatting, playing music, etc. This new virtual medium positively to me was very flexible, convenient, and cost-effective to use. As part of the use of these virtual mediums, I have also acquired some ICT skills following the use of the visual medium platforms.

**R: explain to me your understanding of the virtual medium of education?**

**NS006**: The use of a management system that can either be synchronous or asynchronous to facilitate learning between lecturers and students using the internet.

**R: please share with me the types of virtual learning platforms offered to you by your university?**

**NS006**: Vclass, Zoom meeting, Google meet, Google class, and sometimes WhatsApp. Among all these platforms offered to us, the most preferred was the zoom app because it was easy to use and also synchronous.

**R: please what are your virtual learning experiences?**

**NS006**: My experiences with a virtual medium were both positive and negative. Negatively, I had poor internet connectivity. Power outage is another negative experience I had using the virtual medium. Positively the university increased its data package for students.

**R: please share with me your experiences about the differences between the traditional face to face and the virtual medium**

**NS006**: Data is needed for virtual and not for traditional face-to-face. I could have lectures from a distance with the virtual medium. But with the traditional medium, we students and the teachers must meet in a physical space.

**R: how beneficial is the virtual medium of education to you?**

**NS006**: I didn't need to go to campus for lectures and so I did not need to buy food, pay for accommodation, and transport fairs. It is also very convenient and flexible as I could have lectures anywhere.

**R: what expectations did you have about the virtual medium of education?**

**NS006**: I expected to see my lecturers deliver as they did in the traditional face-to-face. I expected to hear them very clearly using the virtual medium just as we have in the traditional face-to-face. On my part as a student, I expected to have the ability to ask questions and be able to manipulate the virtual medium apps with ease. I also expected lecturers to assess us just like the traditional face-to-face.

**R: please could you describe the extent to which these expectations have been met?**

**NS006:** Most of my expectations were met as I was able to participate actively in the virtual medium with minimal difficulties.

**R: What did you expect with your grades using the virtual medium?**

**NS006:** I expected my grades to improve because taking a quiz or an examination or doing an assignment was very easy as compared to the traditional face-to-face. For example, we could Google for answers for quizzes and also pass on a colleague’s assignment with little modifications as ours. So my grades improved tremendously.

**R: What did you gain from the virtual medium of education?**

**NS006**: I gained some ICT skills. I am also able to manage my time very well as I used the virtual medium.

**R: moving into the future, what do you see as the prospect for the virtual medium of education, especially in Ghana?**

**NS006**: The future is exciting with the virtual medium in Ghana. The flexibility, convenience, and ease of use make the virtual medium a preferred choice for students. So with the government's commitment towards digital their digital agenda, I can say virtual medium has a very bright future in Ghana.

**R: Could you please share with me the challenges encountered using the virtual medium**

**NS006**: The biggest challenge I encountered was the issue of the poor network that affected my internet connectivity. The cost of data also affected some of us with low socioeconomic backgrounds.

**R: Could you please describe the nature of the difficulties you faced using the virtual medium?**

**NS006**: My issue was having to ask for money now and then to be able to take part in the visual medium. I became a nuisance to my parents because of the cost of data on the internet.

R: What were the factors that facilitated your virtual learning?

NS006: The university provided us with a data package to support us in our virtual learning. My laptop never failed me since we started using the virtual medium of education

R: what structures were put in place by your university to assist you in your virtual learning?

NS006: The vclass sometimes could be assessed offline the Wi-Fi at the ICT lab also help us when we got back to campus.

R: Could you please describe the support offered by the university to help you in your virtual learning

NS006: Data package was offered to students to help us in our virtual learning.

R: What are your virtual learning recommendations?

NS006:

R: Is there anything else you will like to comment on about the virtual medium of education that we have not spoken about?

NS006: I recommend the university establish simulation laboratories to take care of the clinical skills lectures which could not be supported using the virtual medium.

R: thank you for your time, God bless you.

NS006: thank you too.

**RESEARCH INTERVIEW TRANSCRIPT**

**IN-DEPTH INTERVIEW WITH NS007**

R: please how old are you?

NS007: I am **23 years old**

R: please, what program are you studying?

NS007: BSc Emergency nursing

R: please tell me your level?

NS007: I am in level 400

Gender: M

R: please what is your religious background?

NS007: I am an Adventist

**R: Please share with me how the covid-19 pandemic impacted your learning?**

**NS007:** The Covid-19 pandemic in general has affected everyone in Ghana. During this period, we were not allowed to hold physical classes on campus. So while off-campus we were introduced to this virtual medium of education. I can say for a fact that without virtual learning the number of months spent could have been wasted. So this virtual learning made me utilize the period of the lockdown whilst at home. Personally, if not for the covid-19 pandemic, I never knew something like zoom meeting or google class. But with the pandemic, I could have lectures from a distance or at home. Before the pandemic, I did not see the complete use of my laptop and smartphone. But when the covid-19 came, I realise the importance of these ICT tools so far as the virtual medium of education is concerned. To be honest with you, The virtual medium was adopted as an emergency intervention following the covid-19 pandemic. So we the students were not ready for it. Mind you, you need some requirements such as computers good internet connectivity, and a stable power source among others to have effective virtual learning. But I guess because we were caught unaware, issues of poor internet connectivity, cost of data, and lack of ICT tools like laptop smartphones affected by virtual learning.

**R: explain to me your understanding of the virtual medium of education?**

**NS007**: Alright with virtual learning I see it as a learning system that is based on formalized and informalized speech using electronic resources. I said formalized because if it is organized between a lecturer and a student it becomes formalized learning. However, it becomes informalized between students themselves. I can also say it is a network transfer of skills and knowledge to recipients at the same time or at different times. For example, I had a situation in that I needed to attend to you and at the same time engage in the virtual medium.

**R: please share with me the types of virtual learning platforms offered to you by your university?**

**NS007**: Ok, the virtual medium platforms we used were vclass, Zoom Google meet Google class, and WhatsApp.

**R: please share with me your virtual learning experiences**

**NS007**: Ok as I indicated the virtual medium came promptly to us. Because of this the good, the bad and the ugly experiences came with the virtual medium of education. To begin with, for instance, I lived in a place where the network is poor. So when we were told we will have lectures via the internet it became a challenge for me. Besides the poor network and internet connectivity, data cost was another issue of concern even though the university provided some support with a data bundle but it was woefully inadequate to meet the demands of the virtual medium of education. For instance, 2.5 gig datum was always given to us for a month before the pandemic Now with the virtual medium, data was increased to 5gig but that could not sustain me let alone a month. Positively the virtual medium helped me manage my time very well. For instance, there was an instance I had to attend an urgent meeting with my landlord and at the same time have zoom lectures. But because the virtual medium permits audio recording I was able to audio record the lectures and attend the meeting as well. However, a painful experience I had was that on this faithful morning, I join a zoom lecture, and halfway into the lecture there was a sudden power outage and that affected me so much.

**R: please share with me your experiences about the differences between the traditional face to face and the virtual medium**

**NS007**: With traditional face-to-face lectures are held in a physical class with both lecturer and students physically present. Also for the traditional face-to-face, I did not need data and internet to attend the class but a virtual medium data bundle for the internet was a basic requirement. One other thing I want to say is that with traditional face-to-face the lecturer can monitor who is actively participating in the class discussion or not. But with the virtual class, attendance and participation become an issue for us. I felt very excited about the traditional face-to-face because I could interact with my colleague's whiles on campus. But with the virtual, it was even difficult to notice the presence of your friends on the virtual platforms. The integrity of our examination using the traditional face-to-face was maintained before the covid-19 pandemic. But with the virtual medium, issues of examination malpractices were recorded among some of us. Also, I realize that with the visual medium of education there were a lot of distractions for me at home. virtual learning is also learning that is self-motivated and so one has to be disciplined to engage in self-paced learning like the virtual medium.

**R: how beneficial is the virtual medium of education to you?**

**NS007**: As I said earlier with this virtual medium, I was able to gain some knowledge on how to manipulate ICT tools such as the PC and headphones as well as the virtual software like the Zoom app. I was able to make good use of the periods of the lockdown with virtual learning. And so months during the lockdown were not lost. Let me also add that this was the most flexible medium of education I have ever encountered. I could take part in the class lectures in the comfort of my home. There was no need to travel to campus for lectures. Initially, because the covid-19 emerged suddenly and we shifted all of a sudden to the virtual medium, I thought it could have affected my grades. But rather my grades improved with the visual medium.

**R: what expectations did you have about the virtual medium of education?**

**NS007**: The first day the virtual medium was mentioned to us following the lockdown, I thought it was going to be free similar to the traditional face-to-face. So I thought all I needed was my laptop or smartphone. I thought we were not going to be using data. besides, I thought once we had gone virtual the university will provide us with stable internet connectivity. I did not believe we could be assessed using the virtual medium. But it happened and was excited about the fact that lectures could be recorded and played at a later date. So with this, I was able to manage my time very well. However, I did not expect that students could cheat on their quizzes and assignments. I got disappointed due to this situation of examination malpractice with the visual medium. One thing that also got me so absurd was that, when we made a shift from the traditional face-to-face to the virtual medium, I thought we could be supported with some ICT tools like laptops, especially for those from less privileged homes.

**R: please kindly describe the extent to which these expectations have been met?**

**NS007**: The internet connectivity, examination integrity, and provision of ICT tools were not met at all since we did not get any support with the above-mentioned concerns. The issue of the cost of data I will say was partially met because the university before the covid-19 pandemic gave students 2.5 gig every month. How the virtual platforms particularly the zoom app operate was excellent.

**R: What did you expect with your grades using the virtual medium?**

**NS007**: Initially I was scared and worried about the visual medium concerning my grades but it rather turns out to be better than I expected.

**R: What did you gain from the virtual medium of education?**

**NS007**: For the virtual medium what I gained in my ability to use my laptop and phone efficiently.

**R: moving into the future, what do you see as the prospect of the virtual medium of education, especially in Ghana?**

**NS007**: As I said we were not ready for the virtual medium of education But with the numerous benefits that came with it despite the challenges I think it is here to stay. What got me disappointed was when students returned to campus after the lockdown the virtual medium usage was halted. So for this university to be part of this digital age regardless of whether there is a pandemic or not the virtual medium should be inculcated into our curricula if we have to join the train of modern education. But I see a good future for virtual learning in Ghana as the government is committed to ensuring the use of ICT in all educational institutions across the country.

**R: Could you please share with me the challenges encountered using the virtual medium?**

**NS007**: All right for the challenges there are myriads of them. As indicated earlier the cost of data was a serious challenge to me even though we were supported by the university with some data. Also, in the place where I lived, I get distracted easily whilst using the virtual medium. At the same, I also encountered challenges with the internet connectivity due to a bad network. Dumsor as is been termed for erratic power supply affected me and many others.

**R: Could you please describe the nature of the difficulties you encountered using the virtual medium?**

**NS007**: Let me talk about the socio-economic nature of my problem. Data for the internet is very costly in Ghana and coming from a very low socioeconomic background, it was very challenging for me to purchase data once I exhaust what is provided by the school. There was a day I have to decide whether to buy data with money meant for foods or use it for data purchases. it was a tough decision but I had to buy it to enable me to participate in the virtual class. Sometimes due to network issues, I may have to move from home to an area where I can get a better network for the internet to be able to partake in the visual class.

R: What were the factors that facilitated your virtual learning?

NS007: A laptop I borrowed from a friend helped me to participate in the virtual medium. There is this school building within my community where I could go to get a better network for my internet. Despite my social-economic challenges, my parents knowing the importance of education were committed to supporting me financially to purchase data for virtual learning. Any time I experience a power outage I fall on friends to record the lectures and send them to me later and this helps me so much. self-motivation was one critical thing that also facilitated my virtual learning. Remember that virtual learning is a kind of self-paced learning that need self-discipline and self-motivation.

R: what structures were put in place by your university to assist you in your virtual learning?

NS007: Although the virtual medium access was free the university complemented our efforts with data. They also made available ICT learning Centres for students to use for their virtual learning activities. IT experts were available to assist students in their virtual learning process.

R: Could you describe the support offered to you by your university using the virtual medium?

NS007: The provision of the data bundle was great support for me. University ICT laboratory was another wonderful initiative by the university to support us.

R: What are your virtual learning recommendations?

NS007: Training for both lecturers and students on the virtual medium will be very helpful going forward. The university together with the government should put in place policies that will make virtual learning less costly or free. Government can also partner with the telcos to reduce the cost of data for students to have an effective virtual education. The virtual medium should be inculcated into our curricula and not wait for pandemics to emerge before we adopt them. Students should be self-motivated and embrace the virtual medium because it has come to stay. Finally, to take care of the skill acquisitions in nursing, simulation labs should be established for the nursing schools.

R: please add anything concerning the virtual medium of education during the covid-19 pandemic that we couldn’t talk about during this interaction?

NS007: I think I have touch-up all the most important areas so far as this conversation is concerned

R: thank you for your time, God bless you.

NS007: Thank you for having me

**RESEARCH INTERVIEW TRANSCRIPT**

**IN-DEPTH INTERVIEW WITH NS008**

R: please how old are you?

NS008: I am **23 years old**

R: please, what program do you study?

NS008: BSc Midwifery

R: please tell me your level?

NS008: I am level 300.

Gender: F

R: please what is your religious background?

NS008: I attend the Assembly of God Church

**R: Please share with me how the covid-19 pandemic impacted your learning?**

**NS008:** The covid pandemic impacted my learning in both negative and positive ways. It was not easy at the beginning with this new virtual medium of education. I am not an independent learner, so I was not a fan of ICT. Because of that initially, things were tough but as we things got better. Studying alone in the house was very difficult for me. Besides the cost of data, poor network and bad internet connectivity created some crises for me in my virtual learning. the virtual medium also created an avenue for laziness and examination malpractices among us students. However, it offered me the opportunity to participate actively by contributing to discussions in class which was difficult for me using the traditional face-to-face.

**R: explain to me your understanding of the virtual medium of education?**

**NS008**: It is a form of teaching and learning with takes over the internet using electronic devices like a laptop and smartphones

**R: please share with me the types of virtual learning platforms offered to you by your university?**

**NS008**: We were offered the vclass, zoom meeting app, Google meet, Google class, zoom app, and sometimes WhatsApp. The zoom was the most preferred because it is simple to use.

**R: please share with me your virtual learning experiences**

**NS008**: I had a lot of experiences with a virtual medium of education. The virtual medium I will say personally did not fit my style of learning. So initially it was not an easy task for me. As I said you need to be self-motivated to learn using the virtual medium. Self-discipline was the order of the day for the virtual medium. coupled with these were the issues data bundle which was very expensive. Poor internet connectivity also compounded the whole virtual medium activities for me. Nonetheless, I was able to acquire some skills resulting from the use of electronic devices and virtual platforms.

**R: please share with me your experiences about the differences between the traditional face to face and the virtual medium**

**NS008**: With the traditional face-to-face I could realize that the participation of students was more active than in the virtual medium because of the physical presence of both students and teachers. The virtual medium I must say it's more flexible a cost-effective than traditional face-to-face as I could have lectures anywhere away from campus.

**R: how beneficial is the virtual medium of education to you?**

**NS008**: It makes learning fun and convenient for me. The energy and time traveling and accommodation, as well as feeding, were all avoided when we started using the virtual medium of education. I also learned some new things about the virtual medium that I did not know.

**R: what expectations did you have about the virtual medium of education?**

**NS008**: I thought I could audio record lectures using all the virtual apps but it was not possible as some of the apps did not support audio recording such as the google meet. I also thought some apps could be operated offline or used without the internet. But I realize that so much data was needed to operate them.

**R: please kindly describe the extent to which these expectations have been met?**

**NS008**: Well I can say my expectations as an outline to you were partially met. This is because the data package was provided to us by the school as well as Wi-Fi for the period we were on campus.

**R: What did you expect with your grades using the virtual medium?**

**NS008**: My grades improved so much but I think it was all because of how loose the virtual medium was concerning assessing the students.

**R: What did you gain from the virtual medium of education?**

**NS008**: I acquired some skills resulting from the manipulation of electronic gadgets. I was also able to manage my time by doing more than one thing at a time.

**R: moving into the future, what do you see as the prospect of the virtual medium of education, especially in Ghana?**

**NS008**: I think the virtual medium is good for us here in Ghana if the problem of the cost of data, and poor internet connectivity among others are resolved. Also if University in collaboration with the government can also provide us with the necessary ICT tools and the training of ICT experts then the virtual medium will be the way to go.

**R: Could you please share with me the challenges encountered using the virtual medium?**

**NS008**: I encountered so many challenges. I had a lot of distractions everywhere at home. I had challenges with the network and internet connectivity among others that came with the virtual medium of education. Data cost also contributed to my inability to take part in the virtual medium at some point.

**R: Could you please describe the nature of the difficulties you encountered using the virtual medium?**

**NS008**:

R: What were the factors that facilitated your virtual learning?

NS008: My parents bought me a phone and a new laptop for this virtual medium.

R: what structures were put in place by your university to assist you in your virtual learning?

NS008: I think the adoption of the v-class and other visual mediums helped me during the covid-19 pandemic. The Wi-Fi provided by the school also helped us in our virtual learning

R: Could you describe the support offered to you by your university using the virtual medium?

NS008: Data package was offered to us to use for our virtual learning. We also had available ICT experts or personnel who assisted us with our technical challenges.

R: What are your virtual learning recommendations?

NS008: I think I have some recommendations for virtual learning. To begin with, the university management needs to train both students and lecturers on the use of the virtual medium. Government should provide the necessary electronic devices to students to enable them to take part in the virtual medium of education. I also recommend that the government establish and refurbish all ICT laboratories in higher institutions of learning to help ground the virtual medium of education. Ghana as a country is bedeviled with erratic power supply, so if the government, as well as the university management, can procure power plants to curb the erratic power supply I think it will help us with the virtual medium of education. Internet data should be subsidized if not free if we want to have a successful virtual medium of education. I will conclude by saying to have a well-grounded virtual learning education ICT should be incorporated into our curricula starting all can you color starting from the basic level to the tertiary institutions.

R: please add anything concerning the virtual medium of education during the covid-19 pandemic that we couldn’t talk about during this interaction?

NS008: I think I have exhausted everything the questions demanded of me

R: thank you for your time, God bless you.

NS008: Thank you for having me

**RESEARCH INTERVIEW TRANSCRIPT**

**IN-DEPTH INTERVIEW WITH NS009**

R: please how old are you?

NS009: I am **24 years old**

R: please tell me your program of study?

NS009: Midwifery.

Gender: F

R: What about your level?

NS009: L400

R: please what is your religious background?

NS009: I attend the church of Pentecost

**R: Please share with me how the covid-19 pandemic impacted your learning?**

**NS009:** Ok the covid-19 pandemic frankly speaking impacted my learning in both good and bad ways. To begin with, the covid-19 pandemic emerges suddenly. This prompted the University to adopt the virtual medium to take care of the emergency. I must say it was a great innovative way to continue education outside of the physical classroom. And this was quite positive for me. The virtual medium though was a smart move made by the university, its sudden adoption and the fact that it was new to us created some problems for me. For instance while at home I had to combine looking after my mum’s provision shop and participate at the same time in virtual learning. This was a daunting task for me for in the beginning individual learning at the same time when was adopted tax me, especially at the beginning. But eventually, I got used to it and I learned new things and gained new knowledge with the use of the visual medium.

**R: explain to me your understanding of the virtual medium of education?**

**NS009**: I see the virtual medium as an electronic form of teaching and learning over the internet.

**R: please share with me the types of virtual learning platforms offered to you by your university?**

**NS009**: We used the vclass, zoom app, Google class Google meet, and WhatsApp. The Vclass, WhatApp, Google class, and Google meet are asynchronous. The zoom app is synchronous and was the most widely used app for lectures between lecturers and students.

**R: please share with me your virtual learning experiences**

**NS009**: I lived in an area where there is a poor network. So internet connectivity was a problem. So I had some challenges with joining the virtual classes and this greatly affected my academic work. Besides, distractions, data costs, and power outages also made virtual learning quite difficult. However, it was a refreshing experience to use the virtual medium despite these challenges. It was frustrating and scary with a lot of anxiety at the beginning but we eventually found our way around the virtual medium of education.

**R: please share with me your experiences about the differences between the traditional face to face and the virtual medium**

**NS009**: I realize that with a virtual medium learning was possible anywhere regardless of the distance but with the traditional medium, you have to travel to campus to take part in lectures using the physical classroom. So with this virtual medium, I could attend lectures in the comfort of my home. The only issue is that I needed good internet connectivity and data to take part in the virtual medium was not easy.

**R: how beneficial is the virtual medium of education to you?**

**NS009:** Ok the virtual medium is beneficial to me in so many ways. It helped me to improve the way I communicate virtually. I am the shy type so I find it difficult to communicate well with my colleagues in class. But with the virtual medium, I could express myself well using the virtual medium. I also gained some ICT skills through the use of the ICT tools like computers and virtual platforms.

**R: what expectations did you have about the virtual medium of education?**

**NS009**: When you covid-19 emerged, and the lockdown was instituted the visual medium was introduced to us. Initially, I had mixed feelings about it. I was excited I could continue my education while at home but at the same time worried about how to use the virtual medium like the zoom app. So my expectations were very low at the beginning because it was a new thing for me. For example, I thought the zoom app was going to be difficult to use but even without pre-training on it, we're able to find our way around it. However, I was expecting the university to provide free internet or provide sufficient data for students to help us in our virtual learning.

**R: please kindly describe the extent to which these expectations have been met?**

**NS009**: Some expectations were met and others were not met. In the case of the cost of data internet connectivity, my expectations were partially met. But my expectations with the use of virtual platforms like the zoom app were met.

**R: What did you expect with your grades using the virtual medium?**

**NS009**: My ability to audio record and listen to lectures later improve my grades tremendously.

**R: What did you gain from the virtual medium of education?**

**NS009**: It improves my ICT skills with the use of electronic devices like laptops, ipads, and smartphones. I also gained some knowledge on how to manage my time since the virtual medium is a self-paced learning medium. Also as I indicated earlier I was able to communicate effectively using the virtual medium better than the traditional face-to-face.

**R: moving into the future, what do you see as the prospect of the virtual medium of education, especially in Ghana?**

**NS009**: I can say the virtual medium is a good innovation for us. I believe if supported will benefit both students and teachers in Ghana, particularly students in nursing. Nursing students can work while they are attending school away from campus.

**R: Could you please share with me the challenges encountered using the virtual medium?**

**NS009**: Ok the challenges are many. Data cost was one of my biggest issues with the virtual medium. The cost of data exerted some economic Burden on me. Also while at home with the virtual medium you get distracted everywhere from family members, vehicle movement, and noises from animals among others. As we all are aware nursing is both a science and an art. The art aspect which deals with the acquisition of clinical skills could not be done using the virtual medium. The nursing department to cater to that. So the virtual was only used for the scientific theories of nursing.

**R: Could you please describe the nature of the difficulties you encountered using the virtual medium?**

**NS009**: There was one time I was using the virtual medium to do my assignment and in the process my data bundle got exhausted. And this affected me so much that day. Even though I got some data later at my own cost, the deadline of the assignment had reached and so I had to pay penalties for late submission and that was a bad experience for me.

R: What were the factors that facilitated your virtual learning?

NS009: The data bundle given to us by the School helped me a lot. I also had a good network that gave me stable internet connectivity.

R: what structures were put in place by your university to assist you in your virtual learning?

NS009: Some virtual mediums such as the vclass, zoom app, Google class, Google meet, and WhatsApp were adopted. The ICT laboratory was also well equipped to help us in virtual learning.

R: What support was offered to you by the university in your virtual learning

NS009: I have some tuition from my colleagues and some lecturers on the use of the visual medium. The data bundle offered to us by the university also supported us a lot in our virtual learning.

R: What are your virtual learning recommendations?

NS009: Government should invest in the virtual medium so that institutions can fully adopt it into their curricula. I also think having simulation Laboratories can help us, especially whilst physical contact is not possible.

R: please add anything concerning the virtual medium of education during the covid-19 pandemic that we couldn’t talk about during this interaction?

NS009: I will like to re-emphasize the need to ensure that these recommendations are followed.

R: thank you for your time, God bless you.

NS009: thank you too.

**RESEARCH INTERVIEW TRANSCRIPT**

**IN-DEPTH INTERVIEW WITH NS010**

R: please how old are you?

NS010: I am **29 years old**

R: please, what program do you study?

NS010: BSc Nursing

R: please tell me your level?

NS010: I am level 400.

Gender: M

R: please what is your religious background?

NS010: I attend the church of Catholic Church

**R: Please share with me how the covid-19 pandemic impacted your learning?**

**NS010:** It severely impacted our learning in the sense that we were locked up in our various homes. We even had doubts and fears as to how education will be continued as far as the curricula activities are concerned. The virtual medium was eventually adopted. Though it was a piece of welcoming news, it also provided numerous challenges to us. It's been good using the virtual medium as we learned a lot. So I will not say it affected me negatively because it came with its benefits.

**R: explain to me your understanding of the virtual medium of education?**

**NS010**: I see it as lectures conducted through a medium outside of the physical classroom

**R: please share with me the types of virtual learning platforms offered to you by your university?**

**NS010**: The university adopted zoom meeting Google meet Google Plus and WhatsApp among others

**R: please share with me your virtual learning experiences**

**NS010**: It's been great it was the first time education was being done on the virtual medium using the internet. I had a great experience. I had the opportunity to use these electronic devices. I meet my lecturers in the comfort of my home. I was able to work around my schedule.

**R: please share with me your experiences about the differences between the traditional face to face and the virtual medium**

**NS010**: With the traditional face-to-face you must be present physically in the class. But with the virtual medium classes are held from a distance over the internet

**R: how beneficial is the virtual medium of education to you?**

**NS010**: It's been beneficial because of how cost-effective, convenient, and flexible it is.

**R: what expectations did you have about the virtual medium of education?**

**NS010**: Initially at the beginning I was nervous because it was a new thing introduced to us. But I realize that similar things were done with the virtual medium just as we have in the traditional face-to-face. So that got me relaxed and moved along with it despite the challenges encountered. I did not expect that the semester could be completed on time or scheduled but with the virtual medium, it became reality.

**R: please kindly describe the extent to which these expectations have been met?**

**NS010**: As I indicated I could not imagine that we will complete the semester, have lectures while at home, and so on. Quizzes, assignments, and examinations could be conducted using these virtual medium platforms. And so I can say my expectations were met using the virtual medium.

**R: What did you expect with your grades using the virtual medium?**

**NS010**: Of course, my grades were much better than when we were using the traditional face-to-face. The visual medium allowed me to learn more so I'll rather recommend virtual media for students and lecturers. It's been fantastic for me to use the virtual medium.

**R: What did you gain from the virtual medium of education?**

**NS010**: I was able to manage my time well. The persistent use of electronic devices and virtual platforms helped me to acquire some ICT skills.

**R: moving into the future, what do you see as the prospect of the virtual medium of education, especially in Ghana?**

**NS010**: Government should pay attention to the telcos concerning data costs for students. I believe the virtual medium is here to stay. The university management should ensure that the internet is available for every student to ensure that virtual learning becomes part of the curricula. The future will be great with all these in place.

**R: Could you please share with me the challenges encountered using the virtual medium?**

**NS010**: The challenges can be seen from the side of the students and faculty. On the side of the students, laziness and lack of discipline are a great distraction for some of us during virtual learning also our electronic gadgets at one point do fillers during the virtual learning on the side of the lecturers he or she may keep postponing lectures but with the treasure face to face the lecturer you don't have the flexibility and time to postpone lectures the biggest challenge is the internet connectivity and the cause of the heater even with the provision of Wi-Fi in the school unless you can have access it means that you must always be ready to buy data to be able to have access to the virtual platform.

**R: Could you please describe the nature of the difficulties you encountered using the virtual medium?**

**NS010**: The nature of my difficulty was the poor network for internet connectivity. I remember, one time in the middle of the night, I had to move from my residence to a secondary school to get a good network for my internet connection to be able to access the virtual platform.

R: What were the factors that facilitated your virtual learning?

NS010: The school's ability to provide us with data and user-friendly virtual platforms was one key thing that made virtual learning is it for me and I also think that our mobile phones and other electronic devices also assisted us so much through the virtual learning process

R: what structures were put in place by your university to assist you in your virtual learning?

NS010: The university adopted some virtual medium platforms which we used for virtual learning. The school wifi also helped us in our virtual education.

R: Could you describe the support offered to you by your university using the virtual medium?

NS010: Even though the university did not provide us with ICT tools or devices like Ipads, laptops, and modems, some data package was provided to help us engage with the virtual medium.

R: What are your virtual learning recommendations?

NS010: How's the country if we want to keep up with the digital Technology driving the economy of the advanced countries like the USA UK China that the government of Ghana must put in place structures to have well-established ICT centers in all instances of higher learning across the country is the internet access should be a priority for the form of government the university management so training should be done for both students a faculty on the use of the natural medium to have a successful implementation of the virtual medium of education besides indiscipline behavior such as absenteeism and noise should be checked by lecturers to curb these issues to make the virtual Media more acceptable

R: please add anything concerning the virtual medium of education during the covid-19 pandemic that we couldn’t talk about during this interaction?

NS010: The badger video should take a bottom-up approach and what I mean is that we must educate ICT right from the basic school through Secondary Education into the tertiary is sushi and everything will be perfect

R: thank you for your time, God bless you.

NS010: Thank you for having me

**RESEARCH INTERVIEW TRANSCRIPT**

**IN-DEPTH INTERVIEW WITH NS011**

R: please how old are you?

NS011: I am **27 years old**

R: please, what program are you studying?

NS011: BSc. Nursing

R: please tell me your level?

NS011: I am in level 300.

Gender: M

R: please what is your religious background?

NS011: I am Muslim

**R: Please share with me how the covid-19 pandemic impacted your learning?**

**NS011:** I'll start by saying that the way the covid-19 pandemic suddenly came has affected my education in both good and bad ways. in a good way it has helped me in learning how to use electronic devices and in a bad way I had to use a new medium of learning unprepared and that affected my learning.

**R: explain to me your understanding of the virtual medium of education?**

**NS011**: Ok so the visual medium of education is a lady medium where a teacher or instructor teaches or shares knowledge with students over the internet.

**R: please share with me the types of virtual learning platforms offered to you by your university?**

**NS011**: So the university gave us the zoom app Google meet, Google class, vclass, and sometimes WhatsApp. I preferred WhatsApp and the zoom app.

**R: please share with me your virtual learning experiences**

**NS011**: Ok so with my experiences as I said earlier, I am not very good at using electronic devices like phones and laptops but even if I do I use them for games, music, and so on. But when the covid-19 pandemic came I realise that is these electronic devices were more useful than just using them for games and music. so with the virtual medium I had education in the comfort of my home using these three devices I was able to have a better way of managing my time and at least was able to acquire some skills with the use of the electronic gadgets.

**R: please share with me your experiences about the differences between the traditional face to face and the virtual medium**

**NS011**: With traditional face-to-face, the lecturer or teacher will be present physically to teach, but virtual teaching and learning occur over the internet. It was also possible to interact with my colleagues after lectures using traditional face-to-face but with the virtual medium, it was difficult. I needed good internet connectivity to be able to hook on to a virtual class which is not necessary with the traditional face-to-face.

**R: how beneficial is the virtual medium of education to you?**

**NS011**: With the virtual medium I was able to carry out my duties at home and yet be able to still take part in virtual learning. Besides it also allowed me to be able to manipulate these electronic gadgets with little difficulty. With the virtual medium, I've been able to acquire some attitude toward learning independently of my colleagues and lecturers because it is a self-paced learning medium.

**R: what expectations did you have about the virtual medium of education?**

**NS011**: I expected to see my teachers and colleagues fully in terms of their physical appearances using the virtual medium. I also expected to be able to use the virtual medium with ease.

**R: please kindly describe the extent to which these expectations have been met?**

**NS011**: My expectations were partially met as I could not see my lecturers fully. Even sometimes to hear my colleagues loud enough was a challenge using the virtual medium.

**R: What did you expect with your grades using the virtual medium?**

**NS011:** Honestly speaking the virtual medium affected my grades so badly. It was very difficult finding my way around these electronic gadgets as well as the use of the virtual apps and I believe that was what affected my grades.

**R: What did you gain from the virtual medium of education?**

**NS011**: I was able to get some skills with the virtual medium using my computer and other ICT gadgets.

**R: moving into the future, what do you see as the prospect of the virtual medium of education, especially in Ghana?**

**NS011**: I think the virtual medium of education is an initiative for students in Ghana if the only government commits resources to it. So that challenges dealing with data costs, accessibility, and ICT tools could be solved, then I think it would be the preferred medium for both teaching and learning for students and lecturers as well.

**R: Could you please share with me the challenges encountered using the virtual medium?**

**NS011**: Yeah so with the difficulties or challenges I can say that at home it was difficult to engage with the virtual medium because of work. It was also difficult to connect to the virtual medium whilst at home for me. The cost data at some point did not allow me to join the zoom lectures.

**R: Could you please describe the nature of the difficulties you encountered using the virtual medium?**

**NS011:** For the period of the lockdown and with the social distancing, it was not possible to have some lectures on the clinical skills acquisition courses. The reason is that the adopted virtual medium platforms did not support demonstration by our clinical instructors. Besides, the school currently does not have any simulation laboratory to assist the students to engage with the clinical instructors following the covid-19 pandemic.

R: What were the factors that facilitated your virtual learning?

NS011: Let me say my laptop and phone help me so much in my virtual learning

R: what structures were put in place by your university to assist you in your virtual learning?

NS011: Well with structures put in place, the fact that we were provided with some virtual platforms for virtual education to me was a very smart initiative on the part of the university.

R: Could you describe the support offered to you by your university using the virtual medium?

NS011: Well for me when I went back to campus I was able to make good use of the ICT laboratory as well as the Wi-Fi which helped me a lot.

R: What are your virtual learning recommendations?

NS011: I think the university should provide the necessary virtual learning requirements such as laptops and iPads as well as modems to help us with virtual learning. I think data could also be subsidized by the university for those of us who cannot afford it. I also recommend that the clinical skills acquisition should be ensured using simulation laboratories

R: please add anything concerning the virtual medium of education during the covid-19 pandemic that we couldn’t talk about during this interaction?

NS011: I think I have said a lot, but I want once more to emphasize the cost data. Something needs to be done about it.

R: thank you for your time, God bless you.

NS011: Thank you for having me

**RESEARCH INTERVIEW TRANSCRIPT**

**IN-DEPTH INTERVIEW WITH NS012**

R: please how old are you?

NS012: I am **36 years old**

R: please, what program are you studying?

NS012: Mphil Nursing

R: please tell me your level?

NS012: I am in level 500

Gender: M

R: please what is your religious background?

NS012: I am a Catholic

**R: Please share with me how the covid-19 pandemic impacted your learning?**

**NS012:** With regards to the covid-19 pandemic I can say it has impacted a lot negatively on my learning, especially during those critical moments of our stay on campus. The covid-19 pandemic has informed the school management to adopt new strategies or platforms for teaching and learning. The adoption has also affected our economic situations as well as affected our physical interaction with colleagues and lecturers in general. I can say that it has negatively impacted our academic work.

**R: explain to me your understanding of the virtual medium of education?**

**NS012**: The visual medium is in my opinion any platform that does not involve the physical contact between students and lecturers but has lectures from a distance using online.

**R: please share with me the types of virtual learning platforms offered to you by your university?**

**NS012**: Yes we were introduced to Vclass and the Zoom meeting app.

**R: please share with me your virtual learning experiences**

**NS012**: When the covid-19 pandemic emerged and the virtual medium was adopted, I became apprehensive. I was wondering what we were going to do to the situation. Because most of us were not prepared for it. So in that regard, I can say it stressed me out. We thought with the virtual medium maybe the school was going to assist us with some electronic gadgets like laptops, tablets, and some amount of data to use. So I can say that my experiences with it were not pleasant. As a developing country, we need to do more to make the virtual medium of education easy for students.

**R: please share with me your experiences about the differences between the traditional face to face and the virtual medium**

**NS012**: In my opinion, there are many differences. With the face-to-face, you are in a physical class with your lectures and colleagues students. So because we are used to the traditional face-to-face, we had difficulties using the virtual medium. Also, the face-to-face lecturers can monitor you and assess whether you participate or not in the class. One other difference I can talk about with the traditional face-to-face is that students aside from meeting in the physical class, they able to develop some soft skills as they socially interact with each other. But with the virtual, you are on your own.

**R: how beneficial is the virtual medium of education to you?**

**NS012**: Alright despite the challenges the virtual medium came with, it is still advantageous to us students. For instance, as students, you may at some point want to have the autonomy to navigate and study what you want to learn. So the virtual medium allows us to do that. Most of the lectures can be recorded and studied later. Depending on the visual medium platform, your contribution in class could be monitored by your teacher.

**R: what expectations did you have about the virtual medium of education?**

**NS012**: Well I can say that what I expected was that after adopting the virtual medium, I expected it to come to stay but I realized that at one point everyone was reverting to the traditional face-to-face. So I thought we could have blended learning instead where some courses are taught using the traditional face-to-face and others using the virtual medium.

**R: please kindly describe the extent to which these expectations have been met?**

**NS012**: Well my expectations were partially met. I was able to use the virtual medium with ease. But I was not happy when some lectures reverted to the traditional face-to-face.

**R: What did you expect with your grades using the virtual medium?**

**NS012**: Expected my grace to be better because the virtual medium offers me the opportunity to re-visit what was thought previously.

**R: What did you gain from the virtual medium of education?**

**NS012**: With the virtual medium, I could manipulate electronic gadgets and virtual medium platforms with ease. It also made me develop an attitude of independent learning.

**R: moving into the future, what do you see as the prospect of the virtual medium of education, especially in Ghana?**

**NS012**: You realize everyone is supporting the virtual medium but the government seems not to show interest in it because you cannot do virtual medium education without proper internet connectivity. So if I have to predict the future of virtual medium education in Ghana, I will say it will not do well if they don't work on certain issues like internet access and data bundle. So until issues of this nature are resolved there is no future for the virtual medium of education in Ghana as I can say.

**R: Could you please share with me the challenges encountered using the virtual medium?**

**NS012**: I had a challenge with getting a laptop. Secondly where I live the network is very poor for internet connectivity. So to join a virtual class I have to travel to another community to be able to access the virtual medium platform. Even in that community once I get there I am confronted with another difficulty of power outages. Besides the risk of road traffic accidents is high in my travels. And then finally the data for the internet was an issue for me. As students, its difficult to get money for feeding talk less of buying data for the internet

**R: Could you please describe the nature of the difficulties you encountered using the virtual medium?**

**NS012**: In search of a good network to have for a virtual class, I nearly had an accident one faithfully day. I just think it was God’s grace.

R: What were the factors that facilitated your virtual learning?

NS012: To me, nothing was done by the university management to facilitate my virtual learning. Students were left to their fate to either adapt to it or not.

R: what structures were put in place by your university to assist you in your virtual learning?

NS012: The only thing I can say with this is the fact that the university management partnered with Vodafone Ghana to give some data to students on monthly basis for the virtual learning I'd also say the library has some computers but not enough to reach the students.

R: Could you describe the support offered to you by your university using the virtual medium?

NS012: Data package was given to us to use in our virtual learning.

R: What are your virtual learning recommendations?

NS012: If the virtual medium is to be maintained and sustained it means that basic computing education should start at the basic level. Secondly, the government needs to immensely invest into the visual medium of education. The university management should partner with the telcos to subsidize the costs of data for internet connectivity to students. The public should be educated on the importance of virtual medium of education so that parents will know what it is and support it

R: please add anything concerning the virtual medium of education during the covid-19 pandemic that we couldn’t talk about during this interaction?

NS012: I think I have spoken all that your questions demanded of me.

R: thank you for your time, God bless you.

NS012: Thank you for having me
